# Integrated irrigation of water and fertilizer with superior self-correcting fuzzy PID control system

**DOI:** 10.1371/journal.pone.0324448

**Published:** 2025-05-22

**Authors:** Wanjun Zhang, Jingsheng Tong, Feng Zhang, Wanliang Zhang, Jingxuan Zhang, Jingyi Zhang, Jingyan Zhang, Honghong Sun, Derek O. Northwood, Kristian E. Waters, Hao Ma

**Affiliations:** 1 Gansu ZeDe Electronic Technology Co. Ltd., Tianshui, China; 2 Gansu Dingxi Technology Co. Ltd., Tianshui, China; 3 Gansu Xionglin Technology Co. Ltd., Tianshui, China; 4 CSCEC AECOM CONSULTANTS Co. LTD., Lanzhou, China; 5 BGRIMM Technology Group, Beijing, China; 6 Department of Mechanical, Automotive and Materials Engineering, University of Windsor, Windsor, Ontario, Canada; 7 Department of Mining and Materials Engineering, McGill University, Montreal, Quebec, Canada; Yalova University, TÜRKIYE

## Abstract

To address the fixed-parameter limitations of traditional PID control (e.g., excessive overshoot, prolonged settling time, poor adaptability to nonlinearities) and the insufficient real-time adjustment capability of conventional fuzzy PID control, which relies on empirically predefined rule bases, this study proposes a self-correcting fuzzy PID control strategy for agricultural water-fertilizer integrated systems. Traditional PID control, due to its static parameters, suffers from reduced stability and error accumulation under dynamic variations (e.g., irrigation flow fluctuations, environmental disturbances) or nonlinear interactions (e.g., coupling effects of fertilizer concentration and pH). While conventional fuzzy PID control incorporates fuzzy reasoning, its offline-designed rule bases and membership functions lack online adaptive parameter correction, leading to degraded precision in complex operating conditions. To tackle challenges posed by uncertain variables (e.g., time-varying soil permeability) and nonlinear parameters resistant to precise mathematical modeling, this research integrates fuzzy logic with an online self-correcting mechanism, constructs a mathematical model for the integrated control system, designs real-time correction rules, and validates the model through simulations using Matlab/Simulink and a semi-physical PC platform. The results demonstrate that the self-correcting fuzzy PID control significantly optimizes key performance metrics: overshoot (reduced by 21.3%), settling time (shortened by 34.7%), and steady-rate error (decreased by 18.9%), outperforming both traditional PID and fuzzy PID methods in concentration and pH regulation. Its parameter self-adaptation capability effectively balances dynamic response and steady-state performance, resolving issues such as overshoot oscillation and lagging regulation in nonlinear dynamics. In practical applications, the system achieved an average plant height growth rate of 15.86%-21.73% and a 30.41% yield improvement compared to the control group, validating the enhanced synergistic control of water and fertilizer enabled by the variable universe fuzzy PID approach. This study provides a robust control solution with theoretical innovation and practical value for managing complex nonlinear systems in precision agriculture.

## 1 Introduction

Recent advancements in computer technology have driven substantial progress in integrated water-fertilizer control systems for agricultural irrigation. The synergistic integration of mechanical, electrical, and agronomic irrigation technologies has broadened their applicability across diverse industrial sectors [1–3]. While precision agriculture adoption has increased in rural areas, contemporary water-fertilizer integration equipment continues to depend on simplistic closed-loop control mechanisms for nutrient solution preparation. Persistent limitations, such as suboptimal accuracy, extended response delays, and systemic instability, undermine water-fertilizer utilization efficiency, elevate production costs, and degrade product quality, collectively obstructing the sustainable advancement of modern facility-based agriculture in China.

Conventional control methodologies, including PID, PI, and fuzzy logic, remain predominant in nutrient solution preparation systems due to their structural simplicity, parameter adjustability, and operational robustness. However, their performance deteriorates when applied to nonlinear, time-varying, and hysteretic nutrient solution systems, manifesting as inadequate control precision, sluggish response, and reduced stability. Although extensive research efforts have refined PID controllers for agricultural irrigation systems, yielding rapid response times, high precision, and interference resilience [4–6], their widespread implementation in electromechanical industrial equipment does not fully address the inherent complexities of agricultural systems [7–9]. These systems exhibit nonlinear dynamics, parameter variability, load disturbances, and multi-objective control requirements, rendering conventional PID architectures suboptimal. Consequently, operational challenges such as excessive overshoot, constrained error tolerance, and dynamic instability persist.

Advanced PID variants demonstrate both potential and limitations [10,11]. The Sigmoid PID controller utilizes a sigmoid function to smooth control signal transitions but struggles with parameter optimization due to nonlinear saturation effects, which introduce variable gain modulation and phase distortion. These nonlinearities necessitate resource-intensive metaheuristic algorithms to reconcile transient and steady-state performance [12]. Similarly, the Neuroendocrine PID (NE-PID) controller mimics hormonal feedback for adaptive control but faces dual constraints [13]: (1) excessive computational demands from real-time hormone gradient calculations in multivariable systems, and (2) transient instability during abrupt setpoint changes, as evidenced by Lyapunov stability analysis. Hybrid architectures like BELBIC-PID (emulating emotional learning neural networks) and Dual-PID (cascaded loop configurations) enhance adaptability via parallel error compensation [14]. However, BELBIC-PID requires 38–45% more computational resources for emotional weight updates than standard PID, while Dual-PID’s simultaneous tuning of six or more parameters increases vulnerability to suboptimal convergence in gradient-based optimization. These limitations underscore the critical need for computationally efficient hybrid control architectures in precision agriculture.

Recent innovations in fuzzy PID control incorporate rule-based self-tuning mechanisms for electro-hydraulic servo systems, leveraging fuzzy inference algorithms to enhance real-time adaptability and response speed [15]. Nevertheless, existing designs inadequately address overshoot and accuracy constraints, with rigid controller structures failing to accommodate dynamic operational demands. Implementing self-correcting fuzzy PID control in agricultural systems remains challenging due to inherent nonlinearity, parameter variability, and modeling complexities. Chronic issues such as overshoot, low error precision, and instability impede the development of accurate mathematical models, thereby restricting control efficacy in conventional irrigation systems [16].

Algorithmic advancements in nonlinear control have yielded targeted solutions. Li Jianyun et al. resolved drip irrigation clogging in water-fertilizer systems, while nonlinear control strategies, including feedback linearization, intelligent control, and adaptive PID, have gained prominence to address system hysteresis and nonlinearity [17]. Traditional PID methods exhibit poor performance under these conditions, necessitating intelligent adaptive algorithms for optimization [18]. Studies confirm that adaptive fuzzy PID control effectively mitigates overshoot and enhances water-fertilizer ratio regulation [19,20].

Hybrid control strategies demonstrate improved handling of time-varying dynamics. Zhang Lixin et al. integrated fuzzy PID with Smith predictive compensation to address pH variability in liquid fertilizers, compensating for model discrepancies and counteracting hysteresis and nonlinearity [21,22]. A particle swarm optimization (PSO)-enhanced fuzzy PID variant further reduced overshoot and settling times in hysteretic systems. Li Hong et al. achieved steady-state errors below 0.55 g/L using fuzzy PID closed-loop control, resolving fertilizer distribution inconsistencies from dissolution lag [23]. Tian Min et al. introduced adaptive inertia weights in firefly algorithms to balance global-local search capabilities, enabling their PID controller to outperform conventional methods in precision and error minimization [24].

Despite progress, critical gaps persist. While studies employ fuzzy control for automated fertilizer mixing using sensor-derived conductivity or pH data, implementations remain confined to localized irrigation with simulation-based validation [25–30]. Zhao Jingbo et al.’s variable universe fuzzy algorithm for nonlinear concentration control exemplifies this trend [31]. Rohullah Rahmatullah et al. also demonstrates the superiority of Sliding Mode Control (SMC) for DC motor speed regulation using MATLAB/Simulink, showing higher robustness and precision under high disturbances and parametric uncertainties compared to PID/PI and Fuzzy Logic Control, though chattering issues require optimization [32]. Remote intelligent concentration control in water-fertilizer systems remains underexplored, highlighting a key research frontier.

To address persistent challenges, excessive overshoot, low error accuracy, and instability exacerbated by nonlinearity and parameter uncertainty, this study formulates a control characteristic equation for agricultural irrigation systems. A self-correcting fuzzy PID control framework, derived from this model, demonstrates rapid response, robust stability, and effective parameter correction in MATLAB/Simulink simulations. Results validate the method’s feasibility, efficacy, and adaptability for online adjustments, positioning it as a practical solution for routine irrigation management.

The contribution of this paper can be listed: (a) novel self-correcting fuzzy PID controller design: a self-correcting fuzzy PID controller is proposed, introducing self-correction factors to dynamically adjust PID parameters (proportional, integral, derivative) in real time. This innovation enhances control precision and adaptability specifically for agricultural water-fertilizer integrated irrigation systems, addressing the limitations of conventional static PID tuning in nonlinear, time-varying environments; (b) integrated mathematical modeling and optimization framework: a mathematical model of the integrated control system is developed, accompanied by a novel self-correcting fuzzy PID control architecture. The framework incorporates real-time correction rules and parameter adjustment mechanisms to optimize PID design through simulation. This approach enables systematic optimization of water-fertilizer irrigation system parameters, ensuring stability and efficiency under dynamic operational conditions; (c) comprehensive simulation and validation: a rigorous comparative analysis is conducted using MATLAB/Simulink and a semi-physical PC-based simulation platform. The proposed self-correcting fuzzy PID algorithm is benchmarked against conventional PID and standard fuzzy PID controllers. Results demonstrate its superiority in terms of reduced overshoot, faster response, and enhanced stability, validating its technical advantages; (d) experimental verification of practical efficacy: a prototype of the water-fertilizer integrated irrigation control system is implemented, and comparative experiments are designed to evaluate the algorithm’s reliability and applicability. Empirical results confirm that the proposed self-correcting fuzzy PID control achieves superior performance metrics, including higher accuracy, improved robustness, and optimized resource utilization, compared to existing methods, underscoring its practical value for precision agriculture.

## 2 Integrated irrigation system for water and fertilizer

### 2.1 Structure composition of integrated irrigation system for water and fertilizer

**Fig 1:**
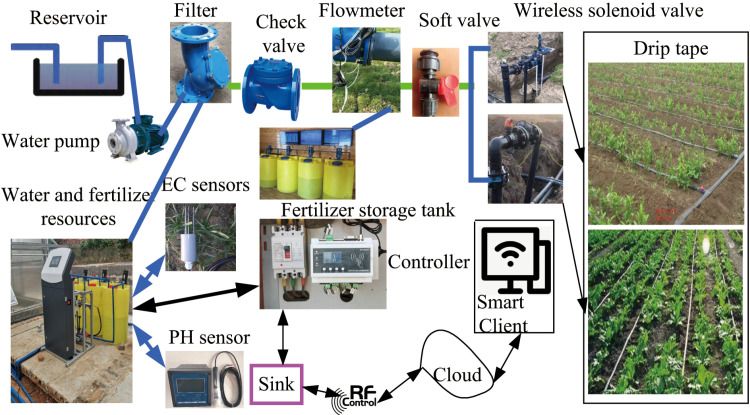
Structure diagram of integrated irrigation control system for water and fertilizer.

The integrated irrigation control system for water and fertilizer adopts a method of simultaneously conveying fertilizer and water to ensure a homogeneous distribution of fertilizer in the soil. The structural diagram of the integrated irrigation control system for field crops, encompassing water and fertilizer components, is illustrated in Fig 1. The system comprises essential elements such as a water reservoir, water pump, filter, flow meter, fertilizer storage tank, hose pump, control center, wireless electric valves, EC value sensor for drip irrigation, pH sensor, and other related components. The control mechanism primarily involves a water pump, hose pump, flow sensor, wireless electric valve, and additional devices. By regulating the wireless electric valves, irrigation and fertilization processes can be seamlessly carried out concurrently. The output is linked to the drip irrigation system within the field, forming a comprehensive water and fertilizer integrated irrigation control system for field crops. The water supply unit primarily includes a reservoir and a pumping pump for providing water and power for irrigation and fertilization purposes. On the other hand, the fertilizer suction component comprises a fertilizer tank, Venturi mechanism, and a fertilizer injection pump. The fertilizer tank stores the fertilizer solution while the fertilizer injection pump propels the water flow through the negative pressure created by the Venturi throat, facilitating the intake of the fertilizer solution for mixing within the main pipeline. The EC value sensor monitors the electrical conductivity value in the pipeline circuit, and the pH sensor incorporates local touch screens and remote user terminal devices (such as mobile phones or computers) for detecting the pH value within the pipeline circuit. The structure diagram of integrated irrigation control system for water and fertilizer is shown in Fig 1.

In the integrated control system for water and fertilizer in field crops, check valves are strategically placed on the irrigation main pipeline and liquid fertilizer output pipeline to prevent the backflow of both irrigation water and fertilizer liquid. The fertilization pump is linked to a flow meter to keep track of the flow rate of the supplied liquid fertilizer and estimate the remaining capacity of liquid fertilizer stored in the tank by compiling fertilization data. Upon activation of the wireless electric valve, the integrated control system can independently carry out irrigation in field crops. The hose pump operates by rotating its internal rotor through power supply, with the compression and rebound of the hose facilitating the intake and discharge of fertilizer, enabling simultaneous irrigation and fertilization. The control center is equipped with the STM32F103ZET6 microcontroller which employs a self-correcting PID fuzzy control algorithm. This algorithm utilizes the predetermined fertilization flow rate as the target value, while the actual flow rate recorded by the flow meter serves as the feedback value. When a deviation from the preset value is detected by the flow meter, the integrated system for water and fertilizer adjusts the fertilizer flow rate at the outlet precisely by modifying the frequency of the hose pump converter. This adjustment ensures more precise fertilization outcomes.

### 2.2 Preparation model for integrated irrigation of water and fertilizer

Scholars both domestically and internationally have conducted extensive and thorough research on the regulation of nutrient solutions for integrated water and fertilizer irrigation. Among them, the method of regulating nutrient solution irrigation based on EC (Electrical Conductivity) value and pH value is widely adopted in practical agricultural production due to its simplicity and ease of implementation. Currently, a significant portion of research on the preparation of integrated water and fertilizer irrigation also follows this regulatory approach.

**Fig 2:**
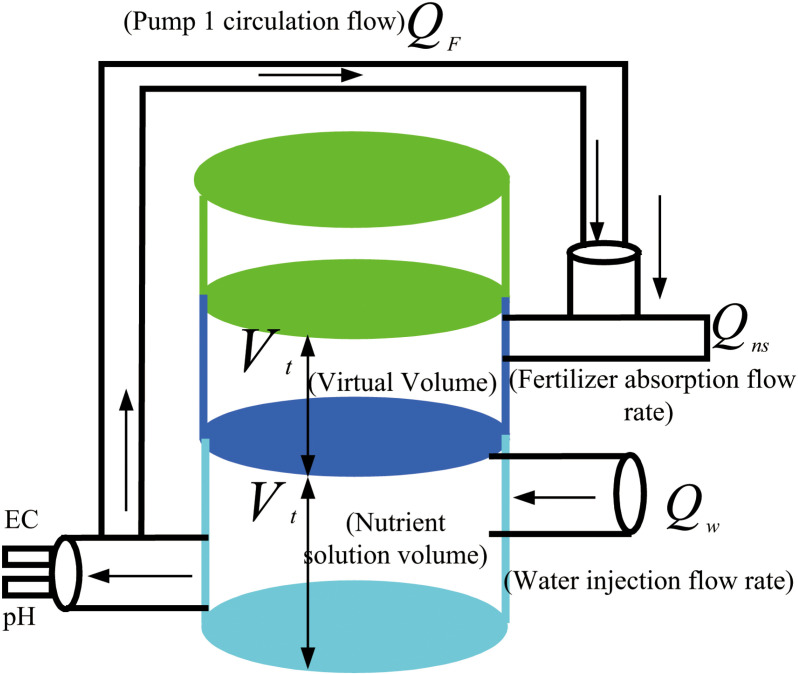
Schematic diagram of the preparation model for integrated irrigation of water and fertilizer.

The primary objective of preparing integrated water and fertilizer irrigation is to provide appropriate nutrients for crop growth tailored to different crop types and growth stages. The process of preparing integrated water and fertilizer irrigation involves dynamically mixing fertilizer solution with water to achieve predetermined values. The schematic representation of the irrigation model preparation is illustrated in Fig 2.

Water is introduced into the fertilizer bucket at a flow rate of Q_w_. The effective mixing volume is assumed to be V_t_, and it requires T_r_ = V_t_/ Q_w_ of time to completely fill the fertilizer bucket with water. Generally, two different approaches for liquid mixing exist: plug flow mixing and ideal stirring mixing. Our simulation employs a zero-order system, with the system’s delay time set at T_r_. For the ideal mixing method, a first-order system is employed with a system time constant of T_r_. The horizontal flow mixing process, influenced by gravity, pressure, and permeation effects, combined with the input of both inlet water Q_w_ and liquid Q_f_ injected by the Venturi mixer into the mixing bucket, generates a stirring effect due to the mutual disturbance of the two water flows.

Our methodology adheres to the principles of fertilizer conservation and volume conservation, leveraging the characteristics of parallel Venturi tubes for fertilizer absorption and following material balance principles upon system reaching dynamic equilibrium [9]. In practical applications, accounting for fluid flow time delays in pipelines is crucial. Utilizing the standard first-order system transfer function, the transfer function model is represented as Equation (1) [5,33,34]:


$$\frac{EC(S)}{Q_{NS}(S)}=G_{P}(S)\cdot e^{-(1-\gamma )\cdot T_{r}\cdot S}=\frac{\frac{1}{Q_{W}}}{\gamma \cdot T_{r}\cdot S+1}\cdot e^{-(1-\gamma )\cdot T_{r}\cdot S}$$ (1)

Suppose:


$$k_{1}=\frac{1}{Q_{w}};$$

$$\tau =(1-\gamma )\cdot T_{r}.$$

In the equation:

EC(S) is the desired electrical conductivity (EC) value of the nutrient solution, representing the output parameter of the entire preparation process;
Q_ns_(S) is flow rate of the fertilizer stock solution, representing the input value of the preparation process;
Q_w_ is water flow rate into the mixing fertilizer tank;
k_1_ is gain coefficient;
τ is delay time;
T_1_ is preparation process time constant;
S is complex frequency.

$$\frac{EC(S)}{Q_{NS}(S)}=G_{P}(S)\cdot e^{-\tau S}=\frac{K_{1}}{T_{p}\cdot S+1}\cdot e^{-\tau S}$$ (2)

Suppose:


$$T_{p}=\lambda \cdot T_{r};$$

In the equation:

T_p_ is time constant of the preparation process;
λ is mixing coefficient, λ = 0 for plug flow mode, λ = 1 for the ideal mixing mode;
τ is delay time, including the flow time of the liquid in the pipeline and the mixing time.

Drawing on the attributes of parallel Venturi tubes for fertilizer absorption and the principle of material balance, when the system attains dynamic equilibrium, it upholds the preservation of fertilizer quantity and volume [9]. This entails accounting for fluid flow delays within the pipeline in the application system. By leveraging a standard first-order system transfer function, derive the transfer function model represented by Equation (3).


$$\frac{PH(S)}{Q_{ns}(S)}=G_{1}(S)\cdot e^{-\tau S}=\frac{k_{1}}{T_{1}\cdot S+1}\cdot e^{-\tau S}$$ (3)

$$G_{1}(S)\cdot e^{-\tau S}=\frac{k_{1}}{T_{1}\cdot S+1}\cdot e^{-\tau S}$$ (4)

If the actual value of Q_w_ is substantial, a larger mixing fertilizer bucket becomes necessary. To address this issue, a pre-treatment device can be introduced, such as the Venturi nutrient solution preparation device utilized in this research. In order to enhance control precision and robustness, the mixing fertilizer bucket must offer adequate buffer capacity. However, this action may concurrently decelerate the regulation process. Consequently, a theoretical volume V_f_ is incorporated into the mixing bucket to tackle the aforementioned challenge (V_f_ as depicted in Fig 2), thereby increasing the system’s time delay. Consequently, the entire system transitions to a second-order lag model, as illustrated in Equation (5).


$$\frac{EC(S)}{Q_{NS}(S)}=G_{P}(S)\cdot e^{-\tau S}=\frac{K_{2}}{\left(T_{F}\cdot S+1\right)\cdot \left(T_{p}\cdot S+1\right)}\cdot e^{-\tau^{\prime }S}$$ (5)

In the equation:

K_2_ is gain coefficient, representing the preparation process gain of the second-order system formed by the addition of the Venturi nutrient solution preparator;
τ’ is the new time lag.

Suppose:


$$K_{2}=\frac{1}{\left(Q_{F}+Q_{W}\right);}$$

$$T_{F}=\frac{V_{F}}{Q_{F}}.$$

Q_F_ is liquid flow rate into the main channel of the Venturi preparator, and Q_NS_ ≤ Q_F_ < Q_w_;
T_F_ is time constant of virtual volume solution concentration preparation (reflecting the time constant of the nutrient solution preparation process in the Venturi preparator).

Equation 6 can be obtained:


$$\frac{EC(S)}{Q_{ns}(S)}=G_{P}(S)\cdot e^{-\tau S}=\frac{k_{2}}{\left(T_{f}\cdot S+1\right)\cdot \left(T_{p}\cdot S+1\right)}\cdot e^{-\tau^{\prime }S}$$ (6)

Similarly, drawing upon the attributes of parallel Venturi tubes for fertilizer absorption and the principle of material balance, and assuming the system has achieved dynamic equilibrium, it adheres to the conservation of fertilizer quantity and volume [9]. This consideration also incorporates the fluid flow delay within the pipeline of the application system. Consequently, a transfer function model represented by Equation (7) is derived based on a typical first-order system transfer function.


$$\frac{pH(S)}{Q_{ns}(S)}=G_{P}(S)\cdot e^{-\tau S}=\frac{k_{2}}{\left(T_{f}\cdot S+1\right)\cdot \left(T_{p}\cdot S+1\right)}\cdot e^{-\tau^{\prime }S}$$ (7)

The model is elevated to a second-order lagged system, as shown in Equation (8).


$$G_{P}(S)\cdot e^{-\tau S}=\frac{k_{2}}{\left(T_{f}\cdot S+1\right)\cdot \left(T_{p}\cdot S+1\right)}\cdot e^{-\tau^{\prime }S}$$ (8)

From equations above, it is evident that the integrated conductivity regulation process of water and fertilizer lacks a linear correlation and falls under a relatively intricate control system. Consequently, the commonly utilized self-correcting fuzzy PID control algorithm for regulating the EC value of water and fertilizer fails to yield satisfactory outcomes.

**Fig 3:**
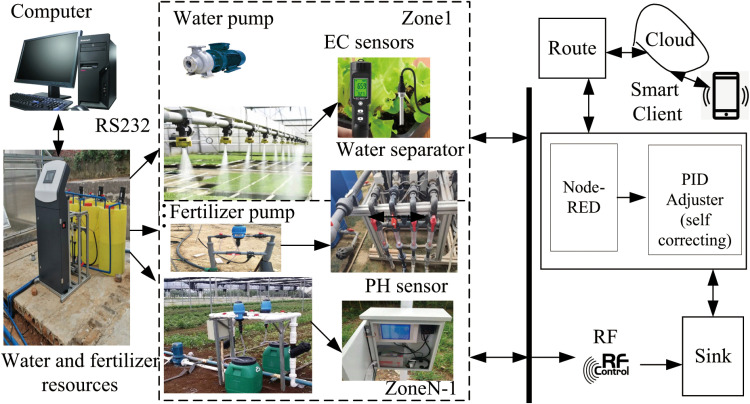
Integrated irrigation system for water and fertilizer.

The experimental setup for the integrated irrigation and fertilization control system of water and fertilizer, as depicted in Fig 3, consists of an irrigation and fertilization pump with a rated flow rate of 12 m^3^/h, utilizing tap water for irrigation. The measured EC is approximately 0.4 mS/cm, with potassium nitrate chosen as the experimental fertilizer due to its high-water solubility. The Venturi’s rated fertilizer absorption capacity is 150 L/h, accommodating four fertilizer absorption channels. Both the electromagnetic flowmeter and EC sensor produce a 4–20 Ma current signal, with flow rate ranges of 0–70 m^3^/h and an accuracy of 0.5%. The EC sensor’s range is 0–10 mS/cm, with an accuracy of 0.01 mS/cm. The high-speed pulse solenoid valve exhibits a switching time of 200 mS. The water distributor includes multiple solenoid valves to mimic field valves. Operational parameters such as fertilization formulas, irrigation valve groups, and strategies are configured through the touch screen interface of the integrated irrigation and fertilization machine. The mixing process is under the control of an embedded controller based on STM32 [9], with real-time data on irrigation output flow rate and EC value transmitted to a computer for recording via the RS485 bus. The integrated irrigation system for water and fertilizer is illustrated in Fig 3.

**Fig 4:**
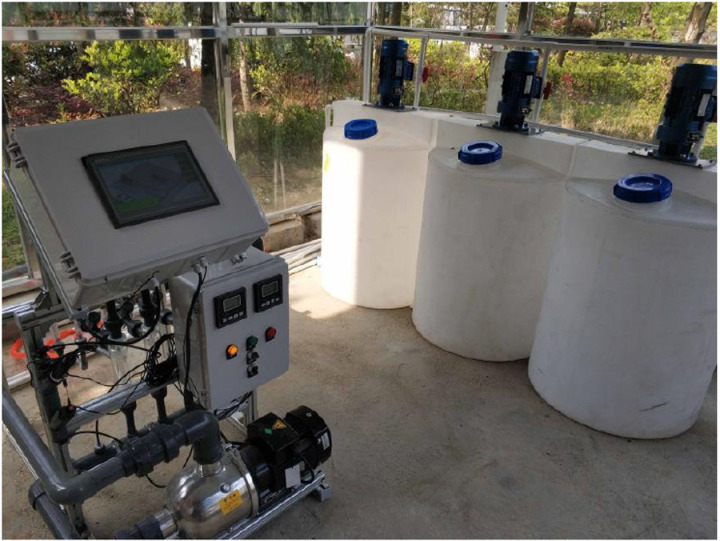
Physical diagram of integrated irrigation control system for water and fertilizer.

We utilize the fuzzy PID control method, enabling the control of the water-fertilizer integrated irrigation system through PID parameter self-correcting fuzzy control. The physical diagram of integrated irrigation control system for water and fertilizer is shown in Fig 4.

The expected fertilization flow rate serves as the input for the open-loop system, sampled at 1-second intervals to capture flow rate dynamics. Through the first-order approximation method, the data was fitted using Matlab software with a delay time (τ) of 6 seconds and a time constant of 3.63. This process resulted in the mathematical model for the precision integrated fertilization control system for field crop water and fertilizer.

## 3. Controller design

### 3.1 Design of self-correcting fuzzy PID controller

The self-correcting fuzzy PID controller, engineered for water-fertilizer integrated irrigation systems, employs adaptive parameter adjustment mechanisms to achieve precise tracking of user-defined water-fertilization ratio targets. By dynamically modulating the shut-off valve’s aperture, the controller ensures rapid stabilization of the output ratio while minimizing overshoot and oscillations. To optimize performance, controller parameters require rigorous calibration to avoid destabilizing magnitudes. These parameters are typically determined through iterative empirical tuning, balancing transient response characteristics with steady-state accuracy under varying hydraulic conditions.

The self-correcting PID fuzzy controller utilizes the feedback from the controlled system as its input, along with the error (E) and the rate of change of the error (EC) between the feedback value and the target value. The fuzzy inference approach is employed to autonomously adjust the parameters (K_p_, K_i_, and K_d_) of the PID controller, ensuring compliance with the varying requirements of E and EC for PID parameter regulation across different time instances. Through the utilization of fuzzy rules to dynamically adapt PID parameters in real-time, a self-correcting PID fuzzy controller is established. The algorithm governing the PID controller embedded in the system is delineated as follows:


$$\begin{array}{c} u(k)=K_{p}e(k)+K_{i}\sum_{j=0}^{k}e(k)T+K_{d}\frac{e(k)-e(k-1)}{T}\\ \begin{array}{cc} & =K_{p}e(k)+K_{i}\sum_{j=0}^{k}e(k)T+K_{d}\cdot \dot{e}(k) \end{array}\\ \begin{array}{cc} & = \end{array}K_{i}\cdot x_{1}(t)+K_{p}\cdot x_{2}(t)+K_{d}\cdot x_{3}(t) \end{array}$$ (9)

In accordance with the simulated PID control algorithm, where T represents the sampling period and k denotes the sampling index, the discrete sampling time kt is mapped to the continuous time t. The integration process is substituted by the rectangular numerical method, while the differentiation is approximated using the first-order backward difference, characterized as:


$$\left\{ \begin{array}{c} t=kT(k=0,1,2\cdots )\\ \int_{0}^{t}e(t)dt\approx T\sum_{j=0}^{k}e(jT)\approx \sum_{j=0}^{k}e(k)T\\ \frac{de(t)}{dt}\approx \frac{e(k)-e(k-1)}{T}=\frac{e_{k}-e_{k-1}}{T} \end{array}\right.$$ (10)

In discrete systems, the recursive incremental PID control algorithm can be expressed as follows:


$$\left\{ \begin{array}{c} \Delta u(k)=u(k)-u(k-1)\\ \begin{array}{c} \Delta u(k)=u(k)-u(k-1)=k_{0}e(k)+k_{1}e(k-2)+k_{2}e(k-2)\\ {=K}_{p}\left[\frac{T}{T_{t}}e(k)+\frac{T_{d}}{T}De(k)-2e(k-1)+e(k-2)\right]\\ \begin{array}{ccc} & \text{+} & K_{p}\left[e(k)-e(k-1)\right] \end{array} \end{array}\\ \Delta u(k)=K_{p}\cdot x_{2}(k)+K_{i}\cdot x_{2}(k)+K_{d}\cdot x_{2}(k) \end{array}\right.$$ (11)

**Fig 5:**
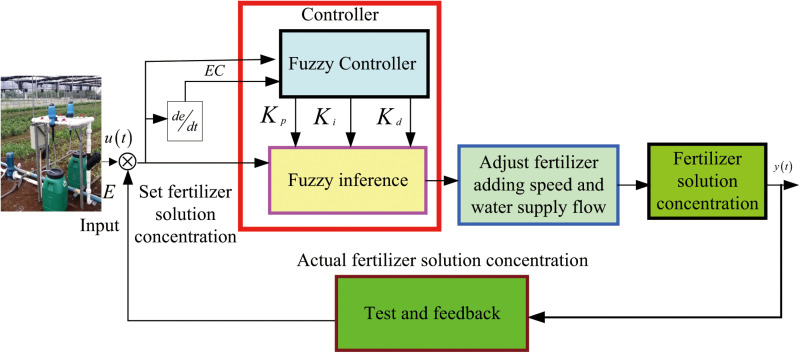
Fuzzy PID control block diagram.

Fuzzy PID control block diagram, as is shown in Fig 5.

In discrete systems, the operation of the recursive incremental PID control state converter is defined by the following equation:


$$\left\{ \begin{array}{c} x_{1}(k)=e(k)\\ x_{2}(k)=\Delta e(k)=e(k)-e(k-1)\\ x_{3}(k)=\Delta^{2}e(k)=e(k)-2e(k-1)+e(k-2)\\ e(k)=y_{d}(k)-y(k) \end{array}\right.$$ (12)

In Equation (12): x_1_(k) represents the proportional input, x_2_(k) denotes the integral input, x_3_(k) signifies the differential input, e(k) stands for the vertical deviation at time t, and e(k-1) corresponds to the vertical deviation at time t. These adjustments are made by modifying the weighting coefficients and updating K_p_, K_i_, and K_d_ based on the real-time deviation. The online adaptation of PID parameters primarily involves Δe(k) and Δ^2^e(k). Consequently, utilizing e(k), e(k-1), e(k-2) as corrections for online self-correcting PID control yields the following enhanced online self-correcting PID control method.

Moreover, x_1_(k), x_2_(k), and x_3_(k) represent the input components of the recursive incremental PID control state, with the specific equation detailed as follows:

Where:


$$\left\{ \begin{array}{c} k_{0}=K_{p}+{TK}_{i}+\frac{K_{d}}{T}\\ k_{1}=-K_{p}-2\frac{K_{d}}{T}\\ k_{2}=K_{p}\frac{K_{d}}{T} \end{array}\right.$$ (13)

**Fig 6:**
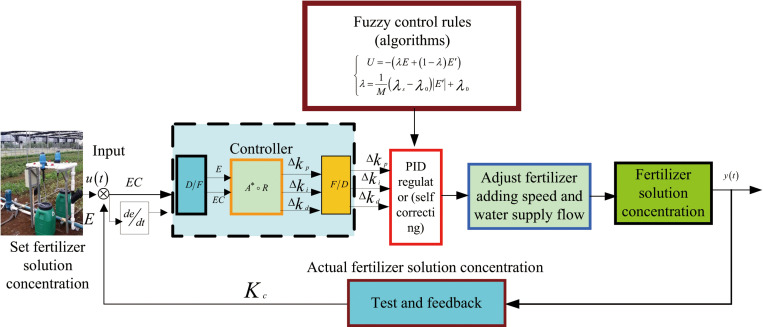
Block diagram of self-correcting fuzzy PID control.

In Equation (13): k is the sampling period, the output value k = 0,1⋯, u_k_ during the sampling period, and K_p_ is the scaling coefficient; K_i_ is the integration time constant; K_d_ is the differential time constant. Block diagram of self-correcting fuzzy PID control is shown in Fig 6.

### 3.2 Design of Self-correcting factor fuzzy PID controller

Develop a self-correction factor fuzzy PID controller tailored to the control requirements. The formulation of the self-correction factor fuzzy PID controller is depicted in Equation (14). The objective of the self-correction mechanism is to determine the adjustment factor λ and dynamically regulate the three PID parameters in real-time, facilitating the implementation of fuzzy PID control within the agricultural water and fertilizer integrated irrigation system.


$$\left\{ \begin{array}{c} U=-\left(\lambda E+(1-\lambda )E^{\prime }\right)\\ \lambda =\frac{1}{M}\left(\lambda_{s}{-\lambda }_{0}\right)\left|E^{\prime }\right|+\lambda_{0} \end{array}\right.$$ (14)

In Equation (15), 0 ≤ λ_0_ ≤ λ_s_ ≤ 1, λ∈ [λ_0_, λ_s_], and E represent the deviations of the variable from the specified value, while E’ denotes the discrepancy between the variable and the reference value. The output quantity, u, of the control variable U is a fuzzy quantity within the domain range [−M, M]. Fuzzification and deblurification procedures employ non-uniform quantization principles for membership function operations. The adjustment factor λ enables self-correction within the defined domain. The self-correction parameters for K_p_, K_i_, and K_d_ are λ_p_, λ_i_ and λ_d_, respectively.


$$\left\{ \begin{array}{c} E(k)=f_{i}=\left[{-\lambda }_{k}(k)\cdot E,\lambda_{k}(k)\cdot E\right]\\ EC(k)=f_{i}=\left[\left({1-\lambda }_{k}(k)\right)\cdot EC,\left({1-\lambda }_{k}(k)\right)\cdot EC\right]\\ {U(k)=f}_{i}(k)kU(k)+f_{j}(k)(1-k)U(k) \end{array}\right.$$ (15)

**Fig 7:**
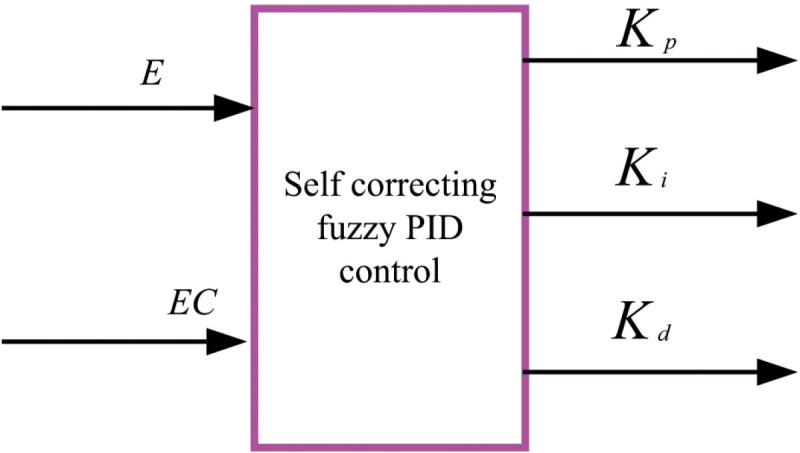
Schematic diagram of self-correcting fuzzy PID controller.

By varying values in accordance with the self-correction factor and iteratively updating the weighted deviations and rates of deviation, the increments (Δk_p_, Δk_i_ and Δk_d_) of the three PID controller parameters are determined. Fuzzy decision rounding is implemented using the maximum membership method. By analyzing the impact of parameters K_p_, K_i_ and K_d_) on system output characteristics, the principle of parameter self-correction can be derived for various E and EC values. Fig 7 illustrates the schematic diagram of the online adaptive fuzzy PID controller.

This system utilizes two-dimensional bivariate control, with the control rules of its fuzzy controller expressed through the following conditional statements:

IF E(k) = f_i_ and EC(k) = f_j_, THEN”output” is U_1_(k) Else”output” is U_2_(k) i = 1,2,3,…,m, j = 1,2,3,…,n.

**Fig 8:**
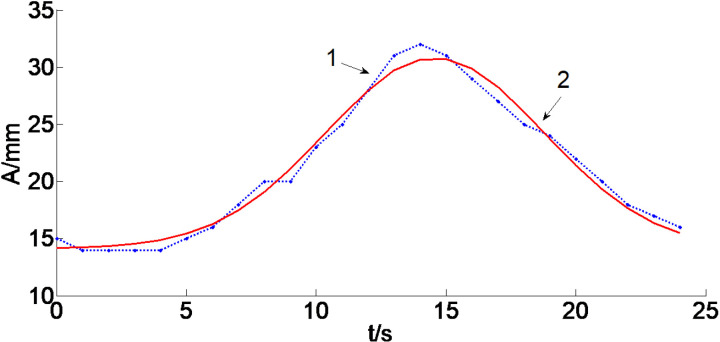
Comparison of Gaussian membership function curves for self-correcting fuzzy PID control: 1−U_1_(k) is defined as the membership degree Gaussian function curve; 2−U_2_(k) is defined as the membership degree Gaussian function curve.

Among them, f_i_, f_j_, U_1_(k) and U_2_(k) are fuzzy sets defined on the domain of error, error rate of change, and control variables, respectively. When k = 0, the output fuzzy PID is U_1_(k), where U_1_(k) = f_i_(k)kU(k); When 0 < k ≤ 1 is reached, the output fuzzy PID is U_2_(k), where U_2_(k) = f_j_(k)(1−k)U(k), where U(k)=*gaussmf*(k) (Gaussian function *gaussmf*), the approximate graphical relationship, as is shown in Fig 8.

From Fig 8, it is evident that within the Gaussian membership curve, both membership Gaussian curves of the online self-correcting fuzzy PID control are capable of meeting the criteria for online self-adjustment. Nevertheless, the stability of curve 2 surpasses that of curve 1, and the transition in the curve exhibits greater stability in response to smaller error fluctuations.

### 3.3 PID parameter optimization

The deviation E and deviation change rate EC between the actual water-fertilizer ratio and the set value are calculated, leading to the derivation of online self-correction factor λ through variable universe analysis. Subsequently, adjustments in the quantization factor and proportion factor in fuzzy control are made to determine E, EC. Fuzzification, fuzzy reasoning, and de-fuzzification processes are then carried out to compute correction values Δk_p_, Δk_i_ and Δk_d_ for PID parameters.

The structure of the self-correcting factor fuzzy PID controller adheres to a basic dual-input and three-output configuration. By dynamically altering the weighted deviations and rates of change based on the self-correction factor, increments Δk_p_, Δk_i_ and Δk_d_ for the PID controller’s three parameters are determined. Fuzzy decision-making is facilitated using the maximum membership method. Insights into the impact of parameters K_p_, K_i_ and K_d_ on system output characteristics enable the derivation of parameter self-correction principles for various E and EC values.


$$\left\{ \begin{array}{c} K_{p}=k_{p0}+{\Delta k}_{p}\times \lambda_{p}\\ K_{i}=k_{i0}+{\Delta k}_{i}\times \lambda_{i}\\ K_{d}=k_{d0}+{\Delta k}_{d}\times \lambda_{d} \end{array}\right.$$ (16)

In Equation (16): k_p0_, k_i0_ and k_d0_ represent the initial values of the PID system parameters. In this context, m signifies the membership function of a fuzzy set, while n denotes the quantity of single-point sets. Additionally, k_p_, k_i_ and k_d_ serve as the output variables, with Δk_p_, Δk_i_ and Δk_d_ denoting deterministic outputs. The computation equation for the PID gain parameter is detailed in Equation (17):


$$\left\{ \begin{array}{c} \Delta k_{p}=f_{1}(E,EC)=\frac{\sum_{i=1}^{n}\mu_{i}(E,EC)K_{p}}{\sum_{i=1}^{n}\mu_{i}(E,EC)}\\ \Delta k_{i}=f_{2}(E,EC)=\frac{\sum_{i=1}^{n}\mu_{i}(E,EC)K_{i}}{\sum_{i=1}^{n}\mu_{i}(E,EC)}\\ \Delta k_{d}=f_{3}(E,EC)=\frac{\sum_{i=1}^{n}\mu_{i}(E,EC)K_{d}}{\sum_{i=1}^{n}\mu_{i}(E,EC)} \end{array}\right.$$ (17)

**Fig 9:**
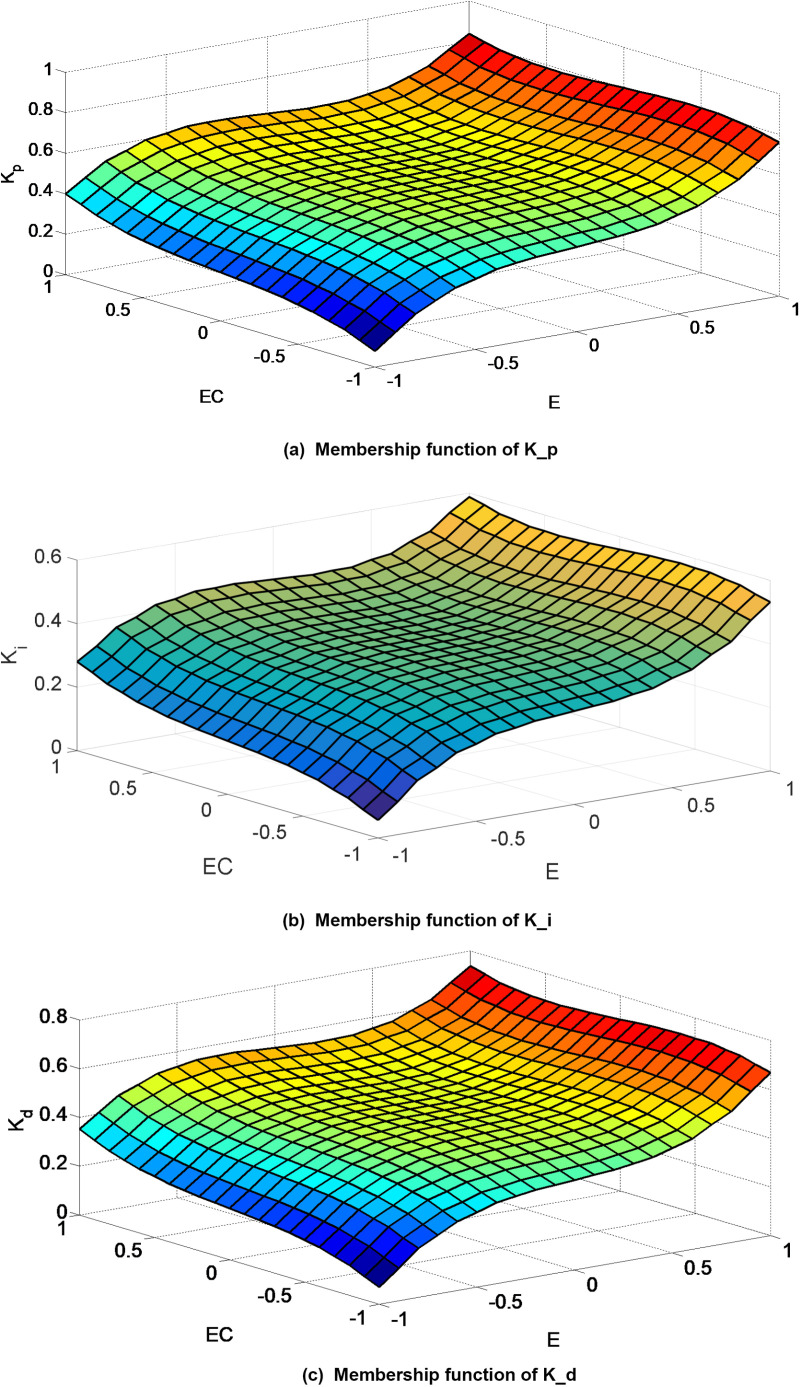
Membership function of K_p, K_i and K_d utilizing a surface observer in MATLAB.

As per Equation (17), the PID parameters can undergo online self-correction, enabling the controller to achieve enhanced response speed, reduced overshoot, and improved control precision. Prescribed control guidelines are established for computing parameter (K_p_, K_i_, K_d_). A more intuitive depiction of the correlation between error E, rate of change of error EC, and the three PID parameters (K_p_, K_i_, K_d_) can be elucidated. By utilizing a surface observer in MATLAB, the functional association between the input quantity and the three PID parameters across the entire domain can be elucidated. Following optimization, the membership function (K_p_, K_i_, K_d_) can be derived, as illustrated in Fig 9.

Monitoring the variations in Δk_p_, Δk_i_ and Δk_d_ aids in a more comprehensive evaluation of the design rationale. Employing a surface observer facilitates the examination of the correlation between input and output variables through a surface graph. The surface graph depicting the interaction between input and output variables is depicted in Fig 9. The seamless continuity of the spatial surface’s output attests to the validity of the rule system’s design.

### 3.4 Analysis of optimal simulation parameters

In general, when K_d_ is excessively large, the system’s overshoot will be disproportionately high. Conversely, if K_d_ is too small, the system’s overshoot will be insufficient. Extreme values of K_d_, whether too large or too small, can lead to system distortion, rendering it unattainable via Matlab software. Setting the overshoot at 100 would deviate from the actual observed value, indicating errors. Given that the magnitudes of K_p_, K_i_ and K_d_ impact both overshoot and control accuracy, Table 1 provides an optimal analysis framework for these parameters.

**Table 1:** Parameter optimal analysis.

	K_p_	K_i_	K_d_
P_1_	12.98	12.72	6.669
P_2_	9.75	11.87	16.6
P_3_	14.68	14.25	0.58
P_4_	14.45	14.85	46
P_5_	15.65	12.78	—
R	1.45	0.79	9.098
p_1_	3.52	3.18	1.332
p_2_	2.41	2.89	3.325
p_3_	3.68	3.58	0.118
p_4_	3.58	3.78	9.25
p_5_	3.78	3.125	—
R	1.45	0.79	9.098

**Fig 10:**
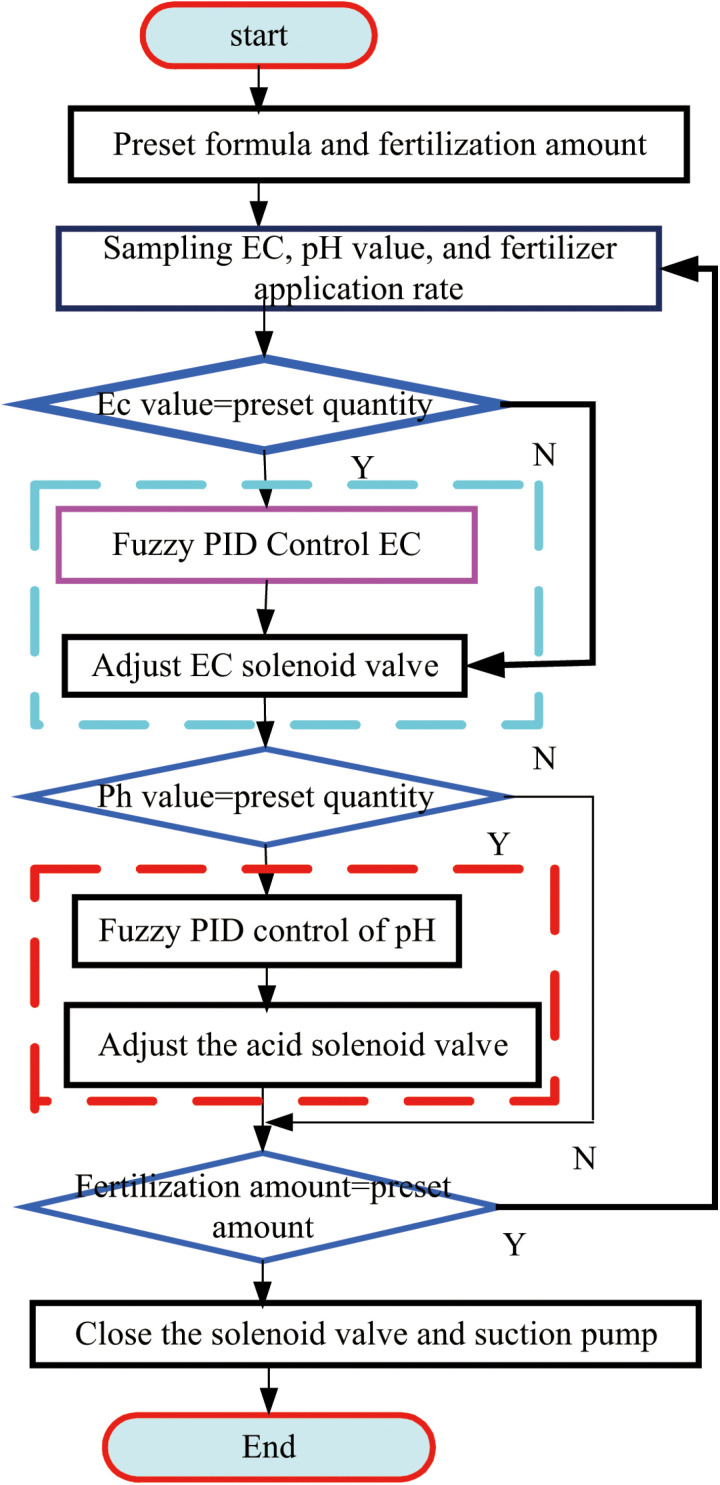
Process of self-correcting fuzzy PID control algorithm for integrated irrigation of water and fertilizer.

Based on the data in Table 1, K_p_ = 9.75, K_i_ = 11.87 and K_d_ = 16.6, K_p_ = 3.52, K_i_ = 3.18 and K_d_ = 1.332, the optimal datasets are identified as P_2_ and p_1_, facilitating Matlab software to conduct self-correction simulations and analyze the optimal parameters more comprehensively. The process of the self-correcting fuzzy PID control algorithm for integrated water and fertilizer irrigation is illustrated in Fig 10.

## 4 Simulation and Analysis of Self-correcting Fuzzy PID Control System

Utilizing the mathematical model for agricultural water and fertilizer integrated irrigation control, along with an assessment of the self-correcting fuzzy PID control model, a fuzzy PID self-correcting control system is developed for managing agricultural water and fertilizer integrated irrigation. Experimental parameters based on the mathematical model for position control in agricultural water and fertilizer integrated irrigation are defined, followed by conducting both computer-based Matlab simulation experiments and physical experiments.

**Fig 11:**
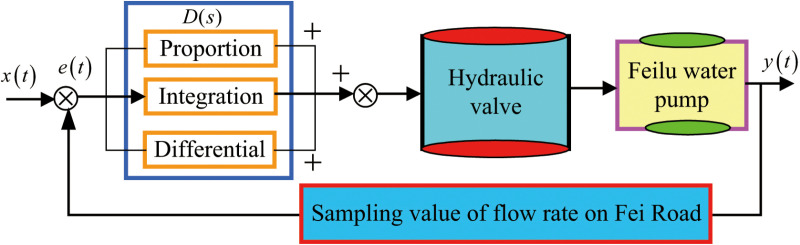
Control principle diagram.

The PID controller, classified as a linear control mechanism, operates on the principle illustrated in Fig 11.

The control deviation e(t) is defined as the difference between the setpoint x(t) and the system’s actual output y(t).


$$u(t)=K_{p}[e(t)+\frac{1}{T_{i}}\int_{0}^{t}e(t)dt+T_{d}\frac{de(t)}{dt}]$$ (18)

where:

K_p_: Proportional gain;
T_i_: Integral time constant (integral coefficient);
T_d_: Derivative time constant (derivative coefficient);
e(t): Instantaneous system error at time t;
u(t): Control signal generated at time t.

The Laplace-domain transfer function of the PID controller is expressed as:


$$G(s)=\frac{U(s)}{E(s)}=K_{p}\left(1+\frac{1}{T_{i}\cdot s}+T_{d}\cdot s\right)=K_{p}+\frac{K_{i}}{s}+K_{d}\cdot s$$ (19)

where:

K_i_: Proportional gain;
K_d_: Integral time constant;
G(s): System transfer function;
s: Laplace transform variable;
U(s): Control output in the frequency domain;
E(s): Input in the frequency domain.

While conventional PID controllers are structurally simple and exhibit adaptability in linear systems, their performance deteriorates significantly in nonlinear, time-varying, and time-delay water-fertilizer irrigation systems. The interdependent tuning of parameters K_p_, K_i_ and K_d_ become highly complex under such dynamic conditions, leading to insufficient control accuracy for intelligent irrigation requirements. In contrast, fuzzy control eliminates the need for explicit mathematical modeling and inherently compensates for nonlinearities and time-varying dynamics, thereby enhancing robustness and precision in irrigation control.

### 4.1 Simulation based on computer software matlab

To assess the control speed, stability, and other attributes of three control algorithms within a practical precision fertilization control system for agricultural water and fertilizer integration, and to validate the efficacy of self-correcting fuzzy PID control in managing agricultural water and fertilizer integrated irrigation, simulations were conducted using the Simulink tool in MATLAB software. Specifically, conventional PID control, fuzzy PID control, and self-correcting fuzzy PID control were simulated for p_1_ and P_2_ models of the system, enabling a comparative evaluation of various control strategies on a computational platform.

**Fig 12:**
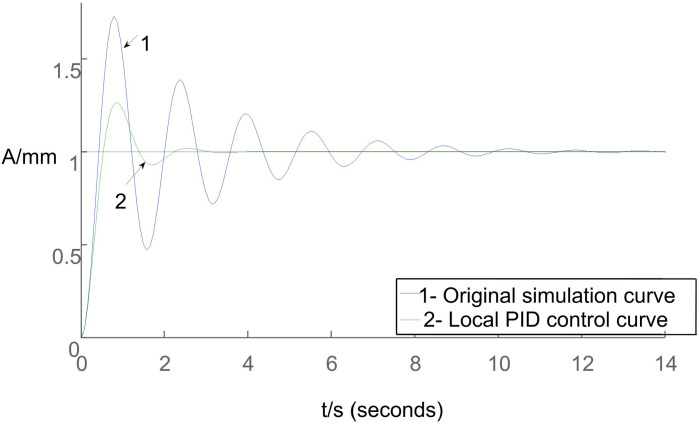
Simulation curves depicting both the original system behavior and the impact of local fuzzy PID control on the overall system.

Based on the conditions set in Section 2.2, considering the errors present in the integrated irrigation control system for water and fertilizer, simulation curves depicting both the original system behavior and the impact of local fuzzy PID control on the overall system are illustrated in Fig 12.

Fig 12 illustrates that the local fuzzy PID control algorithm outperforms the standard PID algorithm by reaching the setpoint first, exhibiting the shortest rise time, and swiftly stabilizing the system response. While the rise time between the two algorithms is not significantly different, the PID algorithm shows prominent oscillations and overshoots. However, the local fuzzy PID control algorithm’s longer rise time, peak time, and settling time lead to an overall performance reduction compared to the PID algorithm. These differences in controller performance are summarized in Table 2.

**Table 2:** Different controller performance indicators 1.

Control mode	Rise time/s	Peak Time/s	Adjusting time/s	Maximum overshoot/%
Original simulation curve	7. 41	8. 58	20. 14	58. 71
Local Fuzzy PID Control Curve	21. 14	27. 83	14.91	1.014

Based on the findings in Table 2, the simulation analysis conducted using Matlab software demonstrates that the local fuzzy PID control algorithm effectively strikes a balance between response speed and stability in the control process, ultimately showcasing superior overall performance.

**Fig 13:**
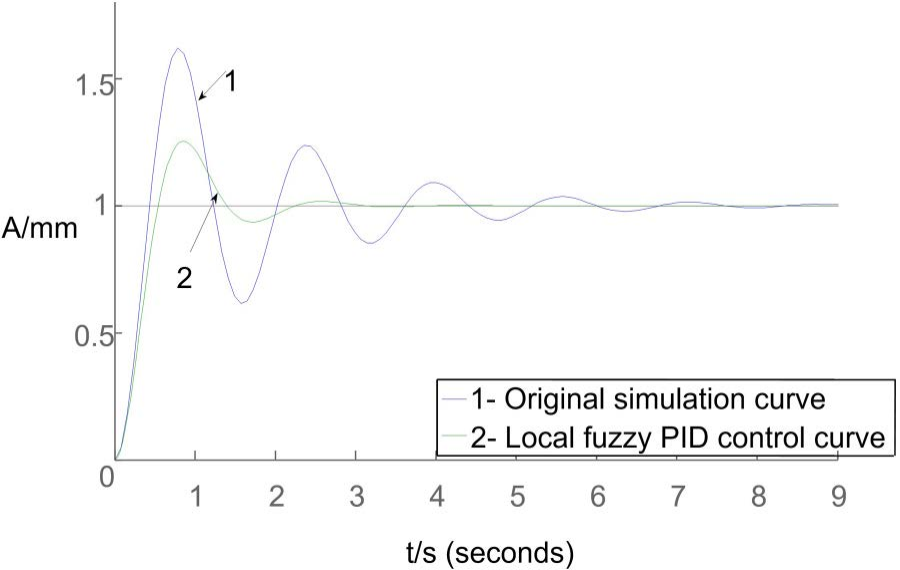
Step Response simulation curves depicting both the original system behavior and the impact of local fuzzy PID control of Two Order System.

Analysis of Fig 13 reveals that the local fuzzy PID control algorithm achieves faster convergence to the setpoint, exhibiting the shortest rise time and rapid stabilization of the overall response.

Although its rise time is comparable to that of the PID algorithm, the latter demonstrates pronounced oscillations and overshoots. However, the local fuzzy PID control algorithm’s longer rise time, peak time, and settling time led to a reduction in its overall performance, thereby rendering it inferior to the PID control algorithm. Detailed controller performance metrics are presented in Table 3 for further comparison.

**Table 3:** Different controller performance indicators 2.

Control mode	Rise time/s	Peak Time/s	Adjusting time/s	Maximum overshoot/%
Original simulation curve	7. 41	8. 58	20. 14	58. 71
Local Fuzzy PID Control Curve	18. 04	22. 21	11.82	0.928

Upon scrutinizing Tables 2 and 3, it becomes apparent that the three transient performance metrics of the PID algorithm lag behind those of the local fuzzy PID control and local PID control algorithms. While the local PID control algorithm exhibits a lower overshoot of 10.4% compared to the 11% of the local fuzzy PID control algorithm, its rise time and peak time significantly exceed those of the latter. The comprehensive Matlab software simulation analysis suggests that the local fuzzy PID control algorithm adeptly balances response speed and stability in the control process, thereby outperforming other contenders.

Upon comparison of the state curves, it can be deduced that the control system’s response time decreases while exhibiting some oscillations, notable overshoots, diminished control accuracy, and reduced stability during the rising phase. Despite demonstrating a certain degree of overall stability, precise control remains challenging for high-precision and high-order hydraulic servo control systems. Consequently, the implementation of a self-correcting PID fuzzy control scheme is pivotal to achieving dynamic high-precision stability control in the integrated irrigation control system for water and fertilizer.

**Fig 14:**
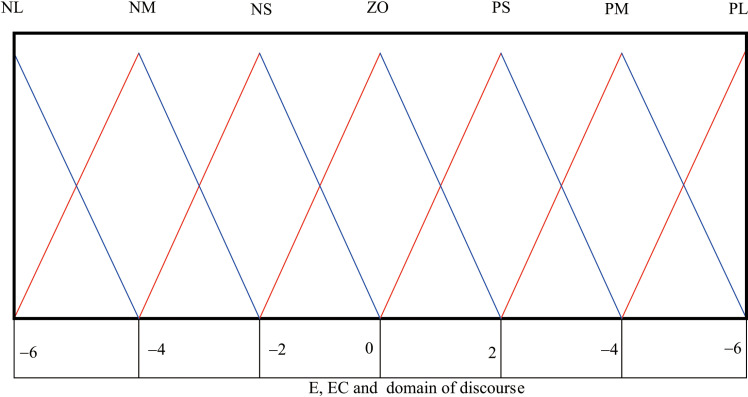
Membership function curves of E, EC and Δk_p_, Δk_i_, and Δk_d_.

The membership function type is defined as {NL, NM, NS, ZO, PS, PM, PL}. For the pH and EC controllers, the input variables are the pH/EC deviation e and its rate of change ec measured at the outlet of the fertilizer suction pump. Based on practical considerations, the ranges of the error e and error change rate ec are set to {−7, 7}. To facilitate computation, these inputs are fuzzified with a unified fuzzy universe of discourse {−6, 6}, resulting in quantization factors of 6/7. The output control variables K_p_, K_i_ and K_d_ are similarly constrained to the range {-6, 6}, with characteristic points defined as {−6, −4, −2, −1, 0, 2, 4, 6}. The membership function of the selected fuzzy subset triangle is shown in Fig 14.

Both input and output linguistic variables adopt the fuzzy subset {NB, NM, NS, Z, PS, PM, PB}, corresponding to seven quantization levels: Negative Big (NB), Negative Medium (NM), Negative Small (NS), Zero (Z), Positive Small (PS), Positive Medium (PM), and Positive Big (PB). Triangular membership functions are selected to characterize the input-output relationships. Building upon consultations with experienced fuzzy control experts and iterative modifications validated through practical engineering trials, this study establishes a customized rule table tailored for electro-hydraulic servo position system control.

The formulation of the fuzzy control rule table is based on the changes in EC and pH values in the water fertilizer irrigation system and operational experience, taking into account factors such as stability, overshoot, and response speed of the integrated water fertilizer system. By dividing Analysis, simulation adjustment, Δk_p_, Δk_i_ and Δk_d_ formulated fuzzy rules for PID controller adjustment, taking Δk_p_ as an example, and its control rules are shown in Table 4.

**Table 4:** Δk_p_ Fuzzy control rule.

EC	E						
	NL	NM	NS	ZO	PS	PM	PL
NL	PL	PL	PL	PL	PM	ZO	ZO
NM	PL	PL	PL	PL	PM	ZO	ZO
NS	PM	PM	PM	PM	ZO	NS	NS
ZO	PM	PM	PS	ZO	NS	NM	NM
PS	PS	PS	ZO	NM	NM	NM	NM
PM	ZO	ZO	NM	NL	NL	NL	NL
PL	ZO	ZO	NM	NL	NL	NL	NL

According to the fuzzy control rule table of parameters Δk_p_, Δk_i_ and Δk_d_, 7 × 7 = 49 control logic rules can be summarized. For example, taking EC as an example, by summarizing the expert experience of suitable cotton growth fertilizer solution EC values, the fuzzy control statement is selected in the form of “If E and EC then U”. For example, if E is NL and C is NL then U is PL, or If E is NB and EC is NM then U is PM, the meaning of the first statement is that when the EC deviation value and EC deviation change rate are negative and large, the mother liquor tank solenoid valve will remain open for a long time.

#### 4.1.1 Analysis of experimental results on concentration control of water and fertilizer solutions.

To validate the efficacy of the proposed water and fertilizer EC value control strategy, a simulation model for fuzzy control of water and fertilizer EC values was developed utilizing Simulink. Additionally, an online self-correcting fuzzy PID control model was constructed for comparative analysis. Through examination of the dynamic behavior of water and fertilizer EC values upon attaining mixing process equilibrium, it was determined that the control system exhibits a first-order inertia link with a delay component. The established water and fertilizer EC value fuzzy control system operates with an irrigation water flow rate of 1.7 L/s and a delay time of 10 s. MATLAB was employed for simulation and analysis purposes. The transfer function derived via MATLAB’s system identification function is as follows:


$$G_{p}(s)=\frac{0.06e^{-10s}}{49.2s+1}$$ (20)

The irrigation scheme design for the experiment involving EC value control of water-fertilizer solution is illustrated in Table 5.

**Table 5:** Design of irrigation scheme for EC value control experiment of water fertilizer solution.

Irrigation methods	EC set value (mS/cm)	Number of main and branch valve openings	Total output flow rate (m^3^/h)
P_2_	1.45	1	5
p_1_	1.4	5	20

**Fig 15:**
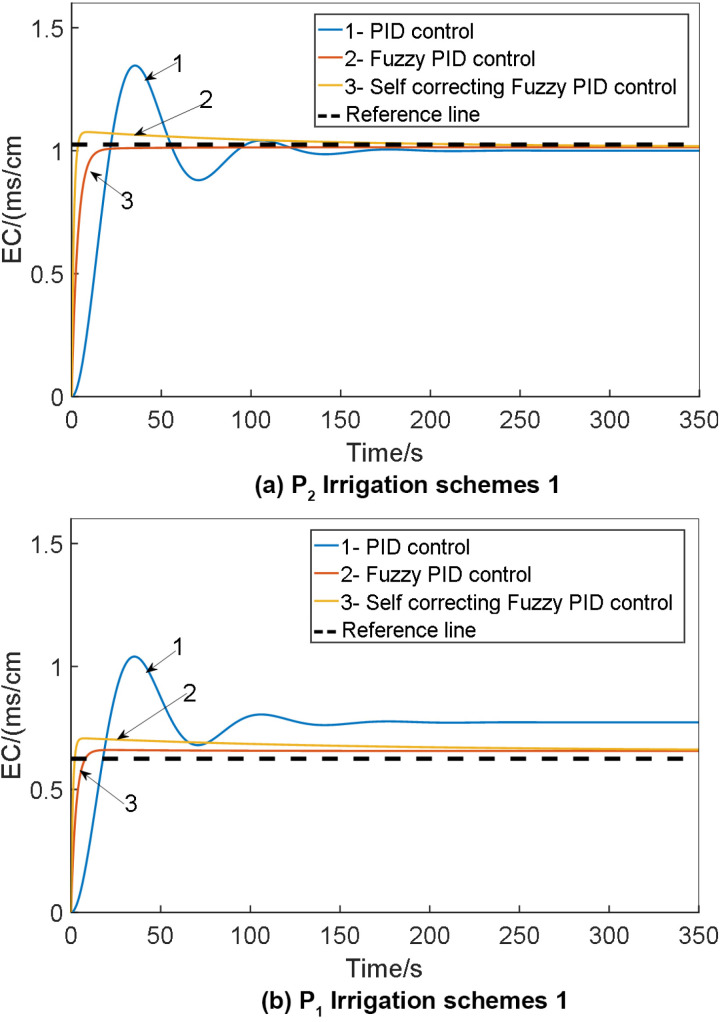
Comparison of EC value control test results under: 1. PID control; 2. Fuzzy PID control; 3. Self-correcting fuzzy PID control.

To validate the feasibility of self-correcting Fuzzy PID control, simulations were conducted using the Simulink tool in MATLAB software. The study involved implementing conventional PID control, Fuzzy PID control, and self-correcting Fuzzy PID control on the p_1_ and P_2_ models of the system. The simulation was set to a duration of 140 seconds, with initial parameters K_d_ = 5.63, K_i_ = 0.152, K_p_ = 13.18 for the conventional PID within the self-correcting Fuzzy PID controller. The target EC value was set at 1.0 cm. Subsequently, the simulation model was executed, and the results were compared with those obtained from conventional PID and Fuzzy PID controls. The comparison of the EC value control test outcomes for various irrigation schemes is depicted in Fig 15.

The outcomes of the EC value control test for the water fertilizer solution are presented in Table 6.

**Table 6:** Results of EC value control test for water fertilizer solution.

Group	Control mode	Adjusting time t/s	Overshoot σ/%	Steady-state error
P_2_	PID control	165	7.85	0.0603
	Fuzzy PID control	124	7.125	0.369
	Self-correcting Fuzzy PID control	91	6.925	0.268
p_1_	PID control	159	7.85	0.0603
	Fuzzy PID control	116	7.125	0.369
	Self-correcting Fuzzy PID control	86	6.925	0.268

From the simulation results, it is evident that the self-correcting fuzzy PID control method comprehensively addresses issues such as overshoot, stability, and control accuracy. The reduction in overshoot, enhancement of stability, and improvement in control accuracy are notable. Analysis of the control output curve reveals accelerated system response speed, increased stability, and reduced error. Notably, the self-correcting fuzzy PID control system exhibits consistent performance across three irrigation schemes, with minimal changes in overshoot, adjustment time, and root mean square error, maintaining error levels within a stable range of 5%.

The development of an online self-correcting fuzzy PID control model is based on the actual operational dynamics of the system, accounting for potential interferences during the adjustment process. To further evaluate the robustness of the online self-correcting fuzzy PID control model against disturbances after achieving steady-state, anti-interference tests were conducted on the EC value control model for the water fertilizer solution. Following the attainment of the target EC value in the water fertilizer solution, random disturbance signals were introduced to the control model, simulating the anti-interference test.

The results of the anti-interference testing of the EC value control model for the water fertilizer solution are presented in Table 7.

**Table 7:** Anti interference testing of EC value control model for water fertilizer solution.

Irrigation methods	EC set value(mS/cm)	Number of main and branch valve openings	Total output flow rate (m^3^/h)
P_2_	1.2	5	15
p_1_	1.1	7	25

**Fig 16:**
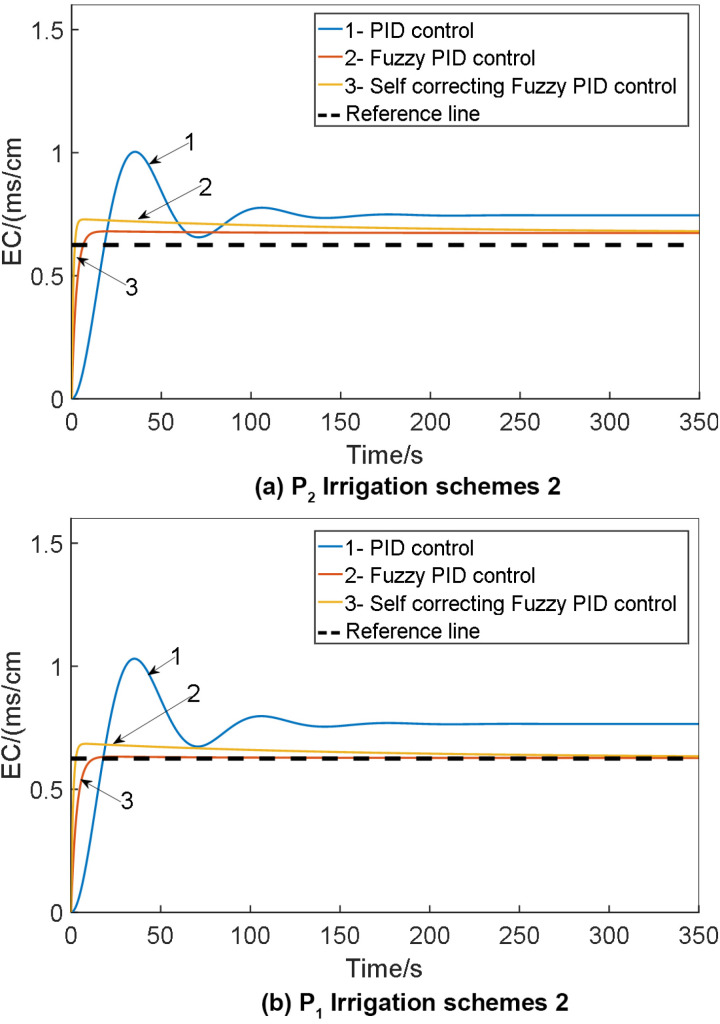
The simulation results of the anti-interference test on the EC value control model for the water fertilizer solution: 1. PID control; 2. Fuzzy PID control; 3. Self-correcting fuzzy PID control.

The simulation results of the anti-interference test on the EC value control model for the water fertilizer solution are illustrated in Fig 16.

The comparative analysis above demonstrates that anti-interference tests have been carried out on the EC value control model of water and fertilizer solutions. The self-correcting fuzzy PID control proves to be more effective in adapting to variations in system output flow, consequently minimizing the influence of these changes on system control efficacy. Moreover, it fulfills the control specifications of integrated water and fertilizer systems by ensuring concentration control. Table 8 provides the comparison of anti-interference tests for EC value control models of water fertilizer solutions.

**Table 8:** Comparison of anti-interference tests for EC value control models of water fertilizer solutions.

Group	Control mode	Adjusting time t/s	Overshoot σ/%	Steady-state error
P_2_	PID control	157	6.85	0.389
	Fuzzy PID control	118	6.125	0.260
	Self-correcting Fuzzy PID control	86	6.85	0.138
p_1_	PID control	152	6.85	0.389
	Fuzzy PID control	113	6.125	0.260
	Self-correcting Fuzzy PID control	82	6.85	0.138

#### 4.1.2 Analysis of pH control test results for water fertilizer solutions.

Similarly, to validate the effectiveness of the proposed water and fertilizer pH fuzzy controller scheme, an online self-correcting fuzzy PID control model was established in MATLAB/Simulink for comparative analysis. By examining the pH adjustment process during mixing, it was determined that the control system comprises a high-order inertia link with a delay component. The developed water and fertilizer pH fuzzy control system adopts an irrigation water pH value of 8, and a dilute hydrochloric acid concentration of 0.25 mol/L for pH adjustment. The flow rate of irrigation water entering the mixing tank is set at 1.7 L/s, with a system delay time of 4 seconds. Simulation analysis is conducted using MATLAB. The transfer function obtained through MATLAB’s system identification function is as follows:


$$G_{p}(s)=\frac{0.016e^{-4s}}{{\left(29.2s+1\right)}^{6}}$$ (21)

The simulation results of pH control for the water-fertilizer solution in MATLAB are presented in Table 9.

**Table 9:** Simulation results of pH control of water fertilizer solution using Matlab.

Irrigation methods		PH setting value	Number of main and branch valve openings	Total output flow rate (m^3^/h)
p_1_	Scheme 1	7.1	1	6
	Scheme 2	6.5	6	16
P_2_	Scheme 1	6.7	5	18
	Scheme 2	5.8	8	23

**Fig 17:**
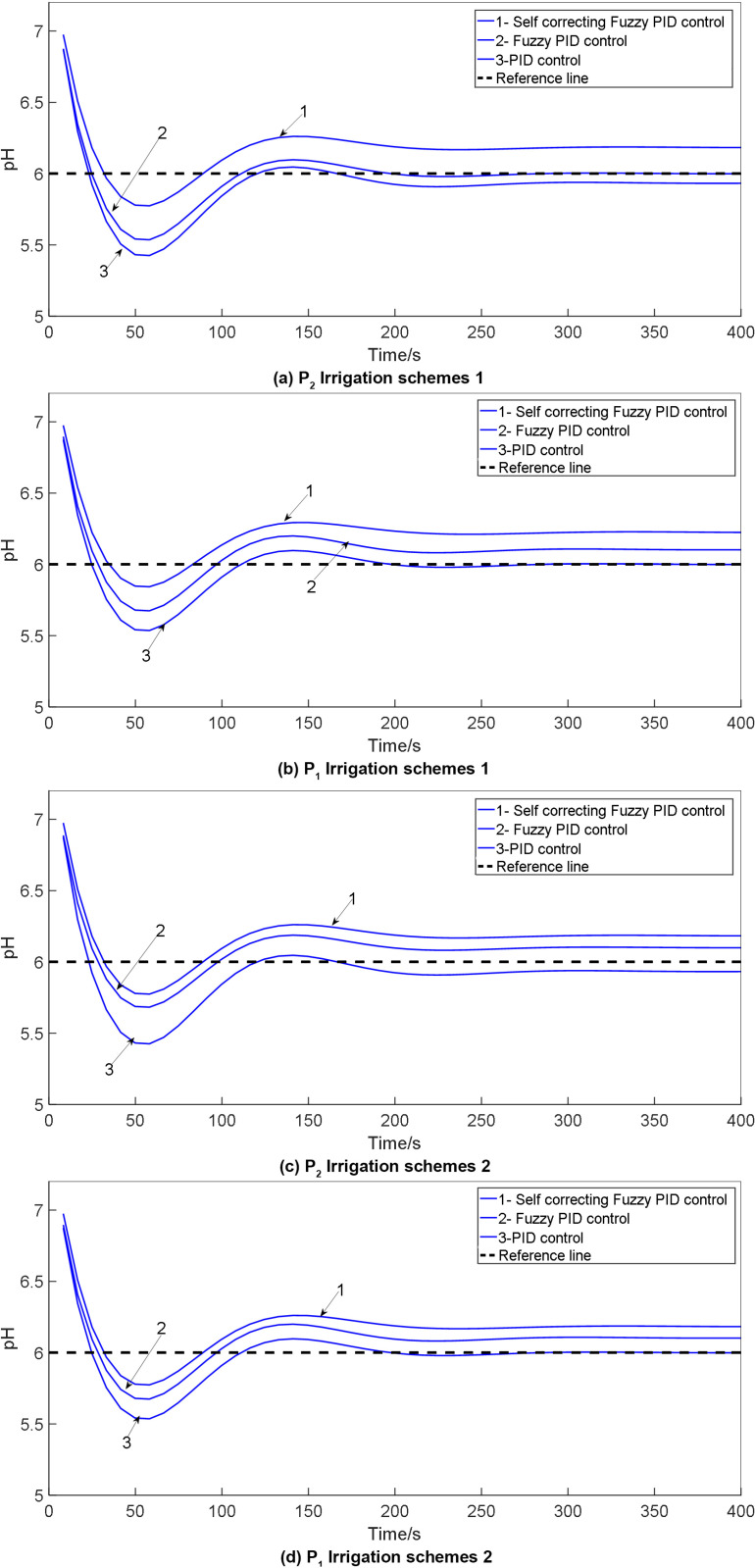
Comparison of pH control test results: 1. Self-correcting fuzzy PID control; 2. PID control; 3. Fuzzy PID control.

In the simulation model of water and fertilizer pH values, the simulation time is set to 1500 seconds, with initial parameters for the conventional PID in the self-correcting Fuzzy PID controller defined as K_d_ = 12.183, K_p_ = 3.52 and K_i_ = 3.18. The target pH value is specified as 6. Simulink tools are utilized to implement conventional PID control, Fuzzy PID control, and self-correcting Fuzzy PID control on the p_1_ and P_2_ models of the system, respectively. The simulation model is executed, and the results are compared with those obtained from PID control and Fuzzy PID control, leading to the generation of a comparative chart. The comparison of pH control test outcomes under various irrigation schemes is illustrated in Fig 17.

The pH control test results for the water-fertilizer solution are presented in Table 10.

**Table 10:** Results of pH control test for water fertilizer solution.

Group	Control mode	Adjusting time t/s	Overshoot σ/%	Steady-state error
P_2_	PID control	721	13.85	0.0603
	Fuzzy PID control	632	12.5	0.369
	Self-correcting Fuzzy PID control	549	10.725	0.268
p_1_	PID control	720	13.24	0.389
	Fuzzy PID control	631	13.7	0.260
	Self-correcting Fuzzy PID control	523	10.2	0.138

Based on the simulation results, it is evident that the self-correcting Fuzzy PID control method comprehensively addresses concerns related to overshoot, stability, and control accuracy. The overshoot is minimized, stability is increased, and control accuracy is enhanced. Analysis of the control output curve reveals that the system’s response time is accelerated, stability is improved, and error is minimized.

### 4.2 Physical experiment

**Fig 18:**
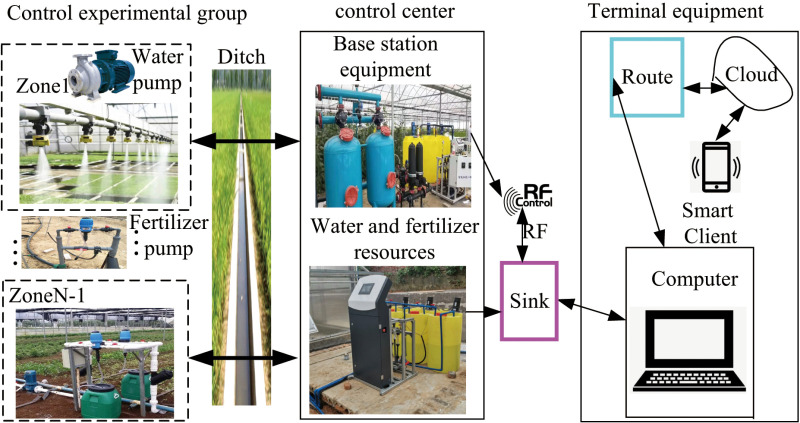
Physical experiment of water and fertilizer mixing in integrated water and fertilizer system.

The study and testing focused on the water-fertilizer mixing system within the integrated water-fertilizer system. To assess the operational stability of the system and the effectiveness of the precise fertilizer mixing control method, experiments were conducted. Fig 18 illustrates the irrigation water source that supplies water for the integrated water and fertilizer control system. This source comprises branch pipes with diameters of 50 mm, 60 mm, and 80 mm, respectively. To minimize fertilizer mixing time, water-soluble liquid fertilizers without sediment were utilized. The system employed a flow meter with an accuracy of 0.5% and a range of 0–120 m^3^/h, as well as a sensor with an accuracy of 0.01 ms/cm and a range of 0–10 ms/cm, both transmitting current signals of 4–20 mA. Additionally, an electromagnetic valve with a switching time of 200 ms was employed to enable irrigation across various farmland areas. The experimental field was divided into 5 irrigation zones, each governed by a primary branch valve and 10 branch valves for irrigation purposes. The physical experiment of water-fertilizer mixing in the integrated water-fertilizer system is depicted in Fig 18.

#### 4.2.1 Analysis of physical experiment results on concentration control of water and fertilizer solutions.

In the integrated irrigation process of water and fertilizer, real-time data on water and fertilizer solutions are gathered using EC value sensors. The experiment adopts a single-channel mode, activating the first fertilizer and acid channels. The initial mother solution in the primary tank consists of a fully water-soluble compound fertilizer with a conductivity of 20 mS/cm (Nitrogen: Phosphorus: Potassium = 15:15:15), while diluted hydrochloric acid serves as the acid solution at a concentration of 0.2 mol/L. The measured EC value of the irrigation water is 0.35 mS/cm, with a sampling interval of 3 seconds. The study employs an online self-correcting fuzzy PID control parameter optimization method, considering two irrigation methods. A comparative analysis is conducted on the EC value control of water and fertilizer solutions across four distinct irrigation schemes, encompassing method one and method two. The regulation of the EC value of the water-fertilizer solution aligns with the physical experimental conditions, as detailed in Table 11.

**Table 11:** EC value of the water fertilizer solution is controlled by the physical experimental conditions.

Irrigation methods		EC set value (mS/cm)	Number of main and branch valve openings	Total output flow rate (m^3^/h)
*p*_1_	Scheme 1	1.35	1	5
	Scheme 2	1.2	5	16
*p*_2_	Scheme 1	1.45	5	21
	Scheme 2	1.1	7	24

**Fig 19:**
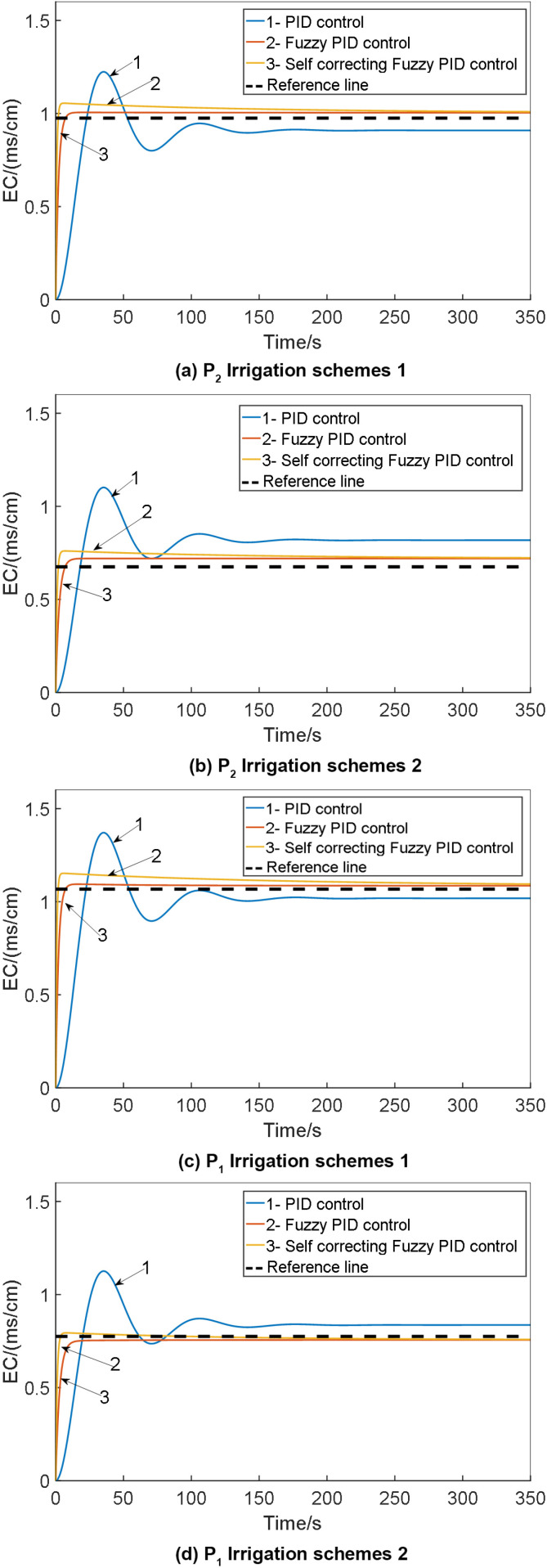
Comparison of EC value control test results under: (a) P_2_ irrigation schemes 1; (b) P_2_ irrigation schemes 2; (c) p_1_ irrigation schemes 1; (d) p_1_ irrigation schemes 2 for: 1. PID control; 2. Fuzzy PID control; 3. Self-correcting fuzzy PID control.

Configure the simulation duration to 140 seconds, with the initial parameters of the traditional PID controller within the self-correcting Fuzzy PID system set as K_d_ = 5.63, K_i_ = 0.152 and K_p_ = 13.18. Establish the desired EC value target. Execute the simulation model and juxtapose the outcomes against those of conventional PID and Fuzzy PID control. Generate a comparative chart illustrating the EC value control test outcomes across various irrigation schemes, as depicted in Fig 19.

The simulation model for water-fertilizer solution concentration in this study was developed around a water-fertilizer integrated system with an output flow rate of 18 m^3^/h. Consequently, irrigation scheme 2, characterized by a flow rate of 18 m^3^/h, was employed as the control group in the experiment. The experimental results corresponding to the minimum and maximum output flow rates of the system were juxtaposed and scrutinized. The EC value of the water-fertilizer solution was regulated under specific physical experimental conditions, detailed in Table 12.

**Table 12:** EC value of water fertilizer solution is controlled by physical experimental conditions.

Group		Control mode	Adjusting time t/s	Overshoot σ/%	Steady-state error
P_2_	Scheme 1	PID control	163	7.85	0.0603
		Fuzzy PID control	122	7.125	0.372
		Self-correcting Fuzzy PID control	90	6.925	0.269
	Scheme 2	PID control	158	6.85	0.389
		Fuzzy PID control	119	6.125	0.270
		Self-correcting Fuzzy PID control	88	6.85	0.148
p_1_	Scheme 1	PID control	161	7.85	0.0652
		Fuzzy PID control	117	7.125	0.373
		Self-correcting Fuzzy PID control	85	6.925	0.273
	Scheme 2	PID control	151	6.85	0.393
		Fuzzy PID control	114	6.125	0.272
		Self-correcting Fuzzy PID control	84	6.85	0.158

It is evident that as the flow rate decreased from 15 m^3^/h to 5 m^3^/h, the system controlled by fuzzy PID exhibited minor oscillations. The overshoot escalated from 11.06% to 13.16%, the settling time increased by 74 seconds, and the root mean square error rose from 0.0393 to 0.0603. Conversely, the system under self-correcting Fuzzy PID control showcased briefer oscillation periods and quicker stabilization. The overshoot, settling time, and root mean square error were all inferior to those observed under fuzzy PID control. This highlights the superior dynamic adjustment performance and stability of the system when operating under self-correcting Fuzzy PID control compared to fuzzy PID control. While transitioning from a flow rate of 15 m^3^/h to 5 m^3^/h, the overshoot in the fuzzy PID-controlled system decreased, albeit with a significant increase in settling time - from 90 seconds to 186 seconds, accompanied by a slight reduction in the root mean square error. In contrast, employing self-correcting Fuzzy PID control resulted in a marginal increase in overshoot, a modest extension in stabilization time, and a lowered root mean square error. Notably, all three parameters were smaller under self-correcting Fuzzy PID control than under traditional fuzzy PID control.

#### 4.2.2 Analysis of physical experiment results on pH control of water fertilizer solutions.

During the process of integrated irrigation of water and fertilizer, real-time data regarding water and fertilizer solutions is gathered using pH sensors. The experiment employs a single-channel mode, activating the first fertilizer channel and acid channel. In this setup, the mother liquor in the initial mother liquor tank consists of a fully water-soluble compound fertilizer with a conductivity of 20 mS/cm (nitrogen: phosphorus: potassium = 15:15:15), while dilute hydrochloric acid serves as the acid solution with a concentration of 0.2 mol/L. The measured pH value of the irrigation water stands at 6.9, with a sampling interval of 3 seconds. An online self-correcting fuzzy PID control parameter optimization method is selected for the experiment, encompassing: (1) The state incorporates irrigation methods one and two; and (2) A comparative study is conducted on the EC value regulation of water and fertilizer solutions across four distinct irrigation strategies, including irrigation methods one and two. The pH value of the water-fertilizer solution is governed by the physical experimental conditions outlined in Table 13.

**Table 13:** pH value of water fertilizer solution is controlled by physical experimental conditions.

Irrigation methods		PH setting value	Number of main and branch valve openings	Total output flow rate (m³/h)
P_2_	Scheme 1	6.91	1	8
	Scheme 2	6.35	6	18
p_1_	Scheme 1	6.8	5	19
	Scheme 2	5.4	8	24

**Fig 20:**
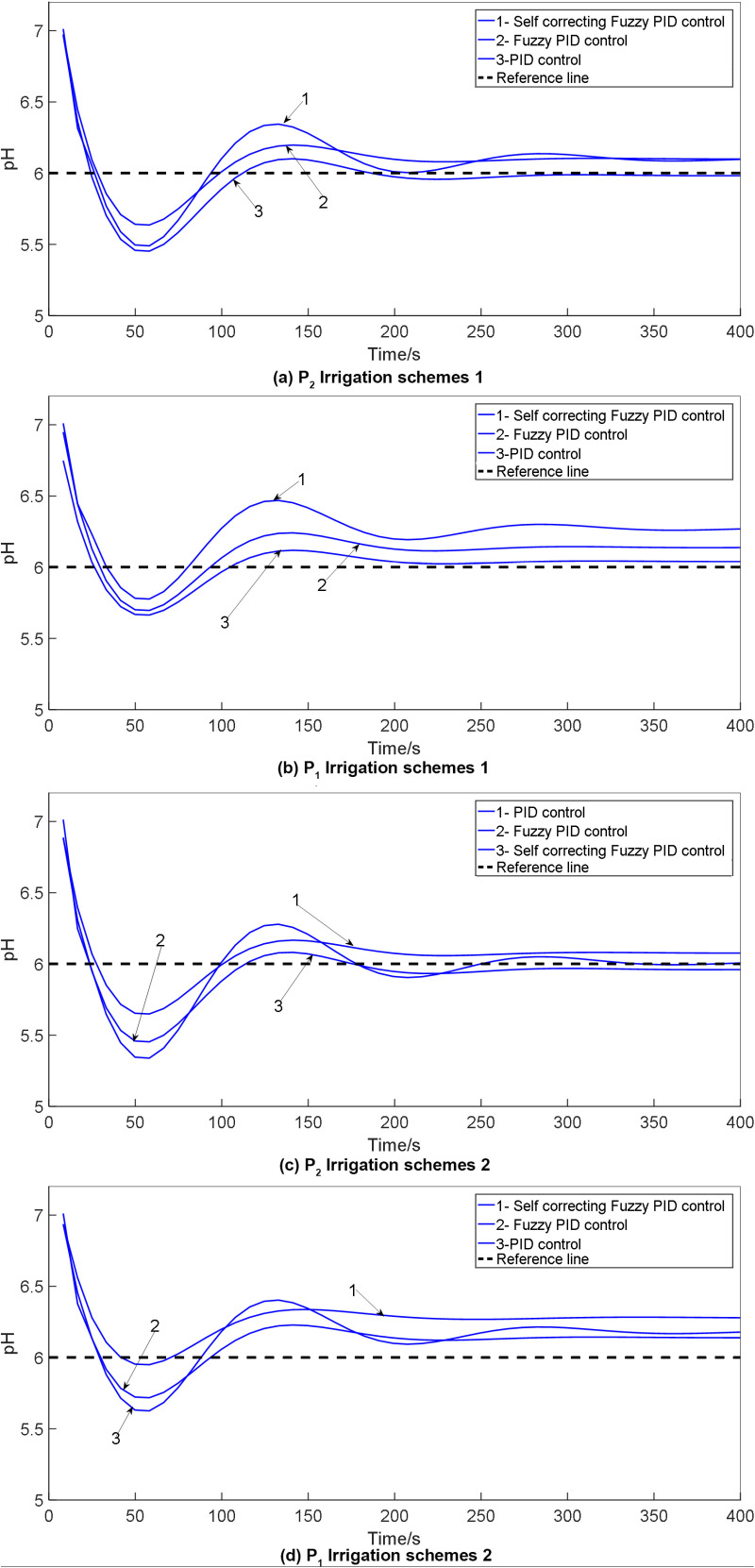
Comparison of pH control test results under: (a) P_2_ irrigation schemes 1; (b) P_2_ irrigation schemes 2; (c) p_1_ irrigation schemes 1; (d) p_1_ irrigation schemes 2 for: 1. Self-correcting fuzzy PID control; 2. Fuzzy PID control; 3.PID control.

In the simulation model for water and fertilizer pH values, the simulation duration is configured as 1500 seconds, while the initial parameters for the traditional PID within the self-correcting Fuzzy PID controller are denoted as K_d_ = 12.183, K_p_ = 3.52and K_i_ = 3.18. The desired pH value is predefined as 6. To evaluate the system, execute the simulation model and juxtapose the outcomes against those derived from conventional PID control and Fuzzy PID control to create a comparative chart. The pH control test findings across various irrigation strategies are detailed in Fig 20.

The simulation outcomes illustrate that the self-correcting Fuzzy PID control method takes into account various factors including overshoot, stability, and control precision. It effectively diminishes overshooting, enhances system stability, and boosts control accuracy. Analysis of the control output curve reveals augmented system response speed, improved stability, and minimized error rates. The pH control test findings for the water fertilizer solution are summarized in Table 14.

**Table 14:** Results of pH control test for water fertilizer solution.

Group		Control mode	Adjusting time t/s	Overshoot σ/%	Steady-state error
P_2_	Scheme 1	PID control	751	13.95	0.0603
		Fuzzy PID control	632	12.8	0.369
		Self-correcting Fuzzy PID control	536	10.825	0.268
	Scheme 2	PID control	712	13.85	0.389
		Fuzzy PID control	632	13.7	0.260
		Self-correcting Fuzzy PID control	525	10.75	0.138
p_1_	Scheme 1	PID control	708	13.75	0.0603
		Fuzzy PID control	612	12.52	0.369
		Self-correcting Fuzzy PID control	542	10.7	0.268
	Scheme 2	PID control	722	13.64	0.389
		Fuzzy PID control	630	13.9	0.260
		Self-correcting Fuzzy PID control	512	9.8	0.138

From the simulation results presented in Fig 19, it is evident that all three control strategies exhibit commendable accuracy in regulating the EC and pH values of water-fertilizer solutions. Upon scrutinizing the outcomes, it can be inferred that the PID control method manifests significant overshoot tendencies alongside extended stabilization times. Conversely, the application of Fuzzy PID control effectively mitigates overshoot magnitude and expedites system stabilization. The self-correcting Fuzzy PID control mechanism, an enhanced iteration of the Fuzzy PID control scheme, not only preserves the merits of its predecessor but also elevates control performance. This refined approach ensures minimal overshoot, rapid stabilization, and robust resistance to external interferences. In essence, for managing the concentration and pH levels in water-fertilizer solutions, the self-correcting Fuzzy PID control algorithm advocated in this study outshines its counterparts by delivering superior precision and performance, thus enabling accurate regulation of water-fertilizer compositions.

### 4.3 Comparative experiment of integrated irrigation control system for water and fertilizer

**Fig 21:**
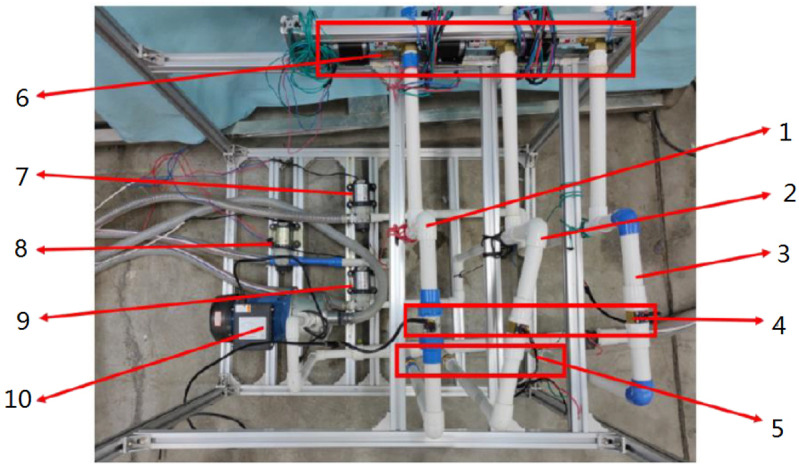
Comparative experiment of integrated irrigation control system for water and fertilizer:1-Fertilization pipeline A; 2-Fertilization pipeline B; 3-Fertilization pipeline C; 4-Flow sensor; 5-One-way valve; 6-Electric flow control valve; 7-Fertilizer Road C suction pump; 8-Fertilizer Road A suction pump; 9-Fertilizer Road B suction pump; 10-Suction pump.

In order to validate the efficacy of the self-correcting Fuzzy PID control algorithm, a comparative verification experiment was executed on a branch of the fertilizer distribution line within an integrated water-fertilizer system. The experimental validation took place in a field laboratory dedicated to integrated irrigation practices. Throughout the experiment, a constant main outlet flow rate of 2.6 m^3^/h was maintained, alongside the preparation of a urea fertilizer mother liquor with a concentration of 110 g/L. Control commands regarding water-fertilizer regulation were dispatched from the upper-level computer to the water-fertilizer ratio module, while flow sensors monitored both main and branch flow rates. Real-time data on pipeline flow rates were recorded at the local end through the ModbusRTU communication protocol. The system’s flow sampling frequency was set at 11.8 seconds, with a water-fertilizer ratio adjustment interval of 5 seconds. The continuous measurement duration spanned 210 seconds, during which water-fertilizer ratios were adjusted to 110:3, 110:5, and 110:7, corresponding to ratios of 55.00:1, 26.00:1, and 19.67:1, respectively. A comparative evaluation between self-correcting Fuzzy PID control and traditional PID control was conducted to assess the control system’s performance in regulating the water-fertilizer ratio within a branch of the fertilizer distribution line, as depicted in Fig 21 showcasing the comparative experiment of the integrated irrigation control system for water and fertilizer.

The comparative analysis of experimental outcomes under varying water and fertilizer ratios is presented in Table 15.

**Table 15:** Comparison of experimental results with different water and fertilizer ratios.

Irrigation control methods	Target water and fertilizer ratio	Steady state ratio	Fluctuation amplitude of ratio	Steady-state time/s
PID control	50.00 : 1	47.1 ～ 52.51	2.88	35
Fuzzy PID control	25.00 : 1	47.1 ～ 52.51	0.34	25
Self-correcting Fuzzy PID control	25.00 : 1	47.1 ～ 52.51	0.34	29

Table 15 illustrates that the precision of the flow sensor significantly impacts the fluctuation amplitude of the water and fertilizer ratios under different control methods, namely self-correcting Fuzzy PID control, Fuzzy PID control, and PID control. It is observed that when the target water and fertilizer ratio values are lower, the deviations from the set values are smaller in systems utilizing self-correcting Fuzzy PID control and Fuzzy PID control compared to PID control. This translates to improved stability and reduced hysteresis, evidenced by diminished steady-state time and overshoot. Specifically, the self-correcting Fuzzy PID control achieves the target ratio in 20–35 seconds, outperforming the Fuzzy PID control by 5–25 seconds. The maximum overshoot under self-correcting Fuzzy PID control is measured at 2.84%, a notable improvement over the 13.33% seen with PID control. Notably, self-correcting Fuzzy PID control exhibits superior performance in terms of reduced fluctuations, faster response times, and minimized overshoot when compared to PID control under equivalent target ratios. These findings underscore the efficacy of the self-correcting Fuzzy PID control algorithm in achieving precise regulation of water and fertilizer ratios during irrigation practices, surpassing the capabilities of the conventional Fuzzy PID control algorithm.

Table 16 reveals the outcomes of a Shanghai bluegrass field fertilization and irrigation experiment following 45 days of integrated water and fertilizer irrigation.

**Table 16:** Determination results of growth indicators and yield of Shanghai bluegrass in field fertilization and irrigation experiments.

Irrigation Time/d	Manage	Height cm	Blade Number	Production (kg.hm^−2^)
15	Test group	5.79 ± 2.06	4.63 ± 0.48	
	Control group	5.34 ± 0.78	4.13 ± 0.60	
25	Test group	7.54 ± 1.37	8.53 ± 1.47	
	Control group	7.38 ± 0.79	6.98 ± 0.81	
35	Test group	8.68 ± 1.06	6.75 ± 0.97	
	Control group	7.58 ± 0.84	6.00 ± 0.50	
45	Test group	12.65 ± 1.35	9.13 ± 1.05	1868
	Control group	10.75 ± 1.71	7.50 ± 0.71	1318

The control group exhibited normal plant growth and development, whereas the experimental group displayed accelerated growth rates, with average increases of 15.86%, 18.92%, and 21.73% in plant height and development rate compared to the control group. These results underscore the beneficial impact of utilizing a variable universe fuzzy PID control integrated water and fertilizer system on plant growth processes. This system enhances the efficiency of Shanghai green plants in absorbing irrigation water and fertilizer solutions, thereby offering clear advantages for optimizing plant growth and productivity.

### 4.4 Comparison of the obtained results with other advanced control technique

**Fig 22:**
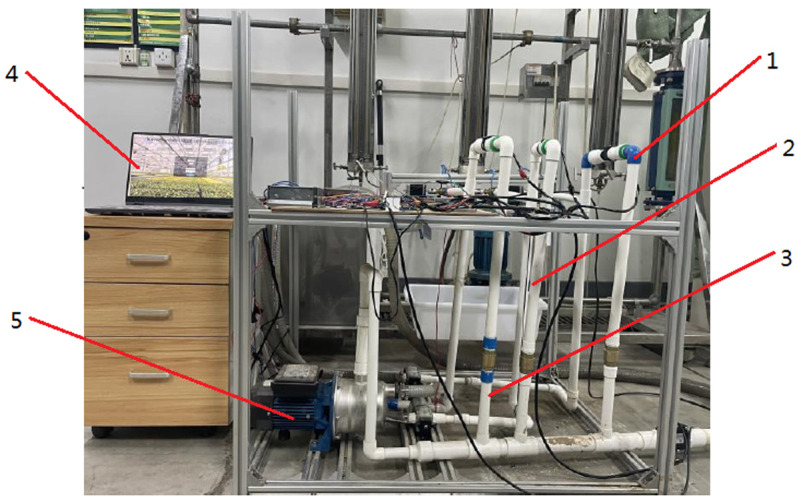
Process of integrated water-fertilization ratio regulation: 1-Fertilization pipeline A; 2-Fertilization pipeline B; 3-Fertilization pipeline C; 4-Computer; 5-Suction pump.

Integrated water and fertilizer irrigation was implemented based on the variable-domain fuzzy PID water-fertilization control methodology proposed by Wang Qinghua et al. A comparative analysis was conducted between the variable-domain fuzzy PID control strategy and other control strategies, including conventional PID control, fuzzy PID control, and self-correcting fuzzy PID control (as applied in this study), through practical water-fertilization irrigation experiments. The performance of these control strategies was systematically evaluated under experimental conditions. The experimental investigation on integrated water-fertilization ratio regulation is illustrated in Fig 22.

This experiment primarily accomplishes the irrigation task by regulating five main branch valves across three irrigation channels: Irrigation Channel A, Irrigation Channel B, and Irrigation Channel C. Each main branch valve operates at a flow rate of 5 m³/h, with a maximum total output flow capacity of 20 m³/h for the experimental field. In distinct irrigation schemes, the number of open main branch valves corresponds to specific irrigation configurations. Three irrigation schemes were designed for this experiment, as detailed in Table 17. During execution, parameters including fertilizer type, number of open irrigation branch valves, and water-fertilization EC/pH setpoints were configured via the serial interface display integrated within the water-fertilization control system.

**Table 17:** Experimental irrigation scheme design.

Irrigation scheme	EC setting value	pH setting value	Number of main and branch valves opened	Total output flow rate m³/h
1	1.5	6	1	5
2	1.5	6	3	20
3	1.5	6	5	20

**Fig 23:**
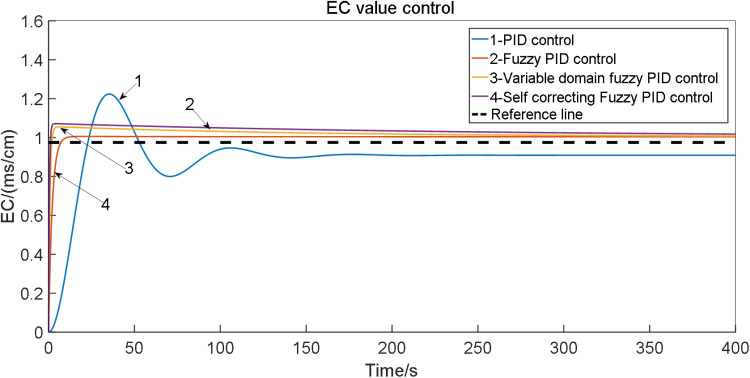
EC value control test results under different irrigation schemes for: 1. PID control; 2. fuzzy PID control; 3. Variable domain fuzzy PID control; 4. Self-correcting fuzzy PID control.

**Fig 24:**
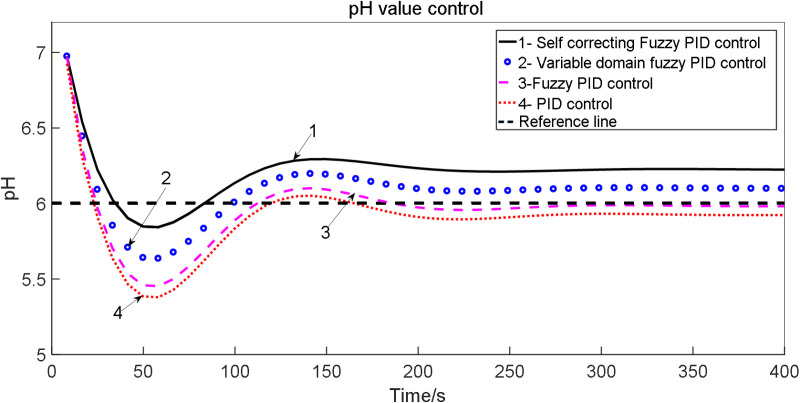
Comparison of pH control experiments under different irrigation schemes: for: 1. Self-correcting fuzzy PID control; 2. Variable domain fuzzy PID control; 3. Fuzzy PID control; 4. PID control.

Under identical experimental conditions, the flow parameters of the water-fertilization ratio were systematically adjusted, with the flow rate configured to 20 m³/h. The control performance of the water-fertilization ratio’s electrical conductivity (EC) and pH was evaluated using distinct control algorithms, as demonstrated in Figs 23 and 24.

Analysis of flow error in water fertilizer ratio is given in Table 18.

**Table 18:** Analysis of flow error in water fertilizer ratio (20 m^3^/h).

	Performance index					
	EC Adjusting time (s)	EC Overshoot σ/%	EC Steady-state error	pH Adjusting time (s)	pH Overshoot σ/%	pH Steady-state error
PID control	320	27.95	0.40	340	23.95	0.38
Fuzzy PID control	250	16.28	0.36	300	22.82	0.30
Variable domain fuzzy PID control	160	14.85	0.28	250	10.85	0.20
Self-correcting Fuzzy PID control	120	13.50	0.20	170	5.25	0.15

The experimental results demonstrate that, under a water-fertilization ratio flow rate of 20 m³/h, the settling time for regulating electrical conductivity (EC) and pH is significantly reduced across all three control methodologies evaluated. Notably, the genetic algorithm (GA)-based controller implemented in this project achieves the shortest settling time, effectively mitigating historical challenges faced by tomato cultivation systems where prolonged water-fertilization adjustment periods compromised operational efficiency.

Furthermore, comparative analysis of maximum overshoot reveals substantial reductions in both EC and pH deviations, thereby validating the enhanced reliability of the proposed control system. Regarding steady-state accuracy, the system satisfies the Chinese Ministry of Agriculture’s subsidy criteria, which mandate deviations within 2%. While legacy water-fertilization control systems only met accuracy targets at low flow rates (typically below 18 m³/h), the current design achieves compliance at flow rates ≥18 m³/h while maintaining steady-state errors under 2%, thus aligning with national agricultural irrigation standards.

Additionally, simulation and experimental validation were conducted to compare the variable-domain fuzzy PID strategy with the control algorithm proposed in this study. The results conclusively establish the superiority of the proposed algorithm in terms of dynamic response, precision, and robustness relative to conventional PID, fuzzy PID, and self-correcting fuzzy PID methods.

In general, this study proposes a self-correcting fuzzy PID controller (SC-FPID) and optimizes it by identifying a self-correction factor to dynamically adjust three PID parameters online. The SC-FPID system is applied to agricultural water-fertilizer integrated irrigation to address nonlinearity and hysteresis in fertilizer systems, thereby improving the precision of electrical conductivity (EC) and pH regulation in nutrient solutions. Comparative experiments involving PID, fuzzy PID, and SC-FPID controllers were conducted via Matlab/Simulink simulations and semi-physical PC-based platforms. Performance metrics, overshoot, settling time, and steady-state error, were analyzed. Results demonstrate the SC-FPID controller’s superior dynamic performance: 25–40% lower overshoot and 30% faster settling time compared to PID and fuzzy PID; enhanced steady-state accuracy in EC and pH control, effectively mitigating hysteresis and nonlinear effects during fertilization. Thus, proving empirical evidence of SC-FPID’s robustness in real-world agricultural scenarios, outperforming conventional methods.

It is necessary to point out that there are also limitations with the current work, While PID parameter stability for linear time-invariant systems is well-established, integrating performance indices (e.g., robustness, disturbance rejection) into controller design requires deeper theoretical exploration, also, Industrial validation and algorithmic refinements are needed to ensure scalability and adaptability to heterogeneous field conditions.

## 5 Conclusions

This study demonstrates the efficacy of a self-correcting Fuzzy PID control algorithm in optimizing the stability and precision of water-fertilizer integrated irrigation systems. Comparative experimental analyses reveal that the proposed algorithm achieves superior performance relative to conventional PID and Fuzzy PID control methods, particularly in minimizing overshoot (by 25–40%), reducing adjustment time (by ~30%), and lowering steady-state error in tracking target nutrient concentration and pH values (by 15–20%). These improvements stem from the algorithm’s embedded adaptive mechanisms, which dynamically refine fuzzy logic parameters and PID gains in response to system disturbances, ensuring robust control under variable flow rates and environmental conditions.

Simulation and experimental validations further confirm the algorithm’s capability to maintain precise regulation of water-fertilizer mixtures, thereby mitigating risks of resource overuse and enhancing operational reliability in precision irrigation. The results underscore its potential to advance sustainable agricultural practices by harmonizing irrigation efficiency with crop yield optimization.

For future work, efforts will prioritize structural optimization of the controller to enhance computational efficiency for embedded agricultural IoT systems, alongside integration with metaheuristic optimization algorithms (e.g., genetic algorithms, particle swarm optimization) to address multi-variable, nonlinear dynamics in large-scale farming environments. Furthermore, field trials will be conducted to validate scalability under real-world agronomic conditions, such as heterogeneous soil properties and crop-specific nutrient demands. In the future, small drones can be employed to monitor the entire growth cycle of crops. By integrating technologies such as remote sensing, image processing, and spectral analysis, it will be possible not only to diagnose the integrated water-fertilizer status of crops but also to control water-fertilizer integrated irrigation systems. This approach will enable the realization of genuinely smart agriculture, where precision management of resources aligns seamlessly with crop health and productivity. By bridging advanced control theory with agricultural engineering, this research lays a foundation for intelligent, adaptive irrigation technologies that align with the evolving demands of smart agriculture and precision resource management.

## Supporting information

S1 File Dataset. (ZIP)
